# Dynamics of bio-based carbon dioxide removal in Germany

**DOI:** 10.1038/s41598-024-71017-x

**Published:** 2024-09-02

**Authors:** Ronja Wollnik, Malgorzata Borchers, Ruben Seibert, Susanne Abel, Pierre Herrmann, Peter Elsasser, Jakob Hildebrandt, Kathleen Meisel, Pia Hofmann, Kai Radtke, Marco Selig, Stanislav Kazmin, Nora Szarka, Daniela Thrän

**Affiliations:** 1 DBFZ-Deutsches Biomasseforschungszentrum gGmbH, Leipzig, Germany; 2 UFZ-Helmholtz Centre for Environmental Research GmbH, Leipzig, Germany; 3 JLU-Justus Liebig University Giessen, Giessen, Germany; 4 UG-University of Greifswald, Greifswald, Germany; 5 TI-WF-Thünen Institute, Hamburg, Germany; 6 HSZG-Zittau/Görlitz University of Applied Sciences, Zittau/Görlitz, Germany

**Keywords:** Carbon dioxide removal (CDR), Negative emissions technologies (NET), Multi-dimensional assessment, Portfolio, Biomass, Climate sciences, Environmental sciences

## Abstract

Bio-based carbon dioxide removal encompasses a range of (1) natural sink enhancement concepts in agriculture and on organic soils including peatlands, and in forestry, (2) bio-based building materials, and (3) bioenergy production with CO_2_ capture and storage (BECCS). A common database on these concepts is crucial for their consideration in strategies and implementation. In this study, we analyse standardised factsheets on these concepts. We find different dynamics of deployment until 2045: for CO_2_ removal rates from the atmosphere, natural sink enhancement concepts are characterised by gradually increasing rates, followed by a saturation and potentially a decrease after few decades; forest-related measures ramp up slowly and for construction projects and bioenergy plants, annually constant removal rates are assumed during operation which drop to zero afterwards. The expenses for removing 1 t CO_2_ from the atmosphere were found to be between 8 and 520 € t CO_2_^−1^, which arises from high divergence both in capital and operational expenditures among the concepts. This high variability of expenses seems to suggest the more cost-effective concepts should be implemented first. However, aspects from economics, resource base and environmental impacts to social and political implications for Germany need to be considered for developing implementation strategies. All concepts investigated could be deployed on scales to significantly contribute to the German climate neutrality target.

## Introduction

Carbon dioxide removal (CDR) describes a range of human-induced actions that capture carbon dioxide (CO_2_) from the atmosphere and store it durably^1^. Although it is widely agreed that climate protection primarily requires a reduction of greenhouse gas emissions, there is large consensus on the need for CDR to reach international and national climate targets^2^. In both international and national climate strategies, CO_2_ removal is regarded as a complementary measure that can contribute along with substantial and immediate reduction and avoidance of greenhouse gases^3^. CDR methods produce net negative emissions if the greenhouse gas emissions from applying the method (upstream and downstream emissions as well as CO_2_ leakage) are less than the CO_2_ removed from the atmosphere. For the purpose of this study, a minimum storage duration of 100 years is regarded as permanent storage.

The German Climate Protection Act sets the target of greenhouse gas neutrality by 2045, and net negative emissions after 2050^4^. Several national climate neutrality studies have calculated the implications for the national carbon budget^5–10^. According to these studies, by mid-century German residual emissions are estimated to be between 45 Mt CO_2_eq a^−1^^7^ and 80 Mt CO_2_eq a^−1^^8^ from agriculture, industry, and other sectors. Thus, the results of the studies indicate that CDR is indispensable to achieve net-zero emissions by 2045.

A range of CDR options is available to reach these ambitious targets. While they might have great potentials, all come with risks and disadvantages. Knowledge on these has to be constructed in a systematic and comparable way with the aim of implementing targeted regulations that take into account local dynamics^11,12^.

According to Ref.^13^, CDR methods are categorised into biological, geochemical and chemical, based on the way the CO_2_ is removed from the atmosphere. Virtually all of the current CDR is biological, coming mainly from afforestation and forest management. A small fraction comes from novel CDR methods such as bioenergy with carbon dioxide capture and storage (BECCS) and biochar^3^. Carbon storage in bio-based CDR takes different forms after the initial CO_2_ uptake from the atmosphere through photosynthesis. Firstly, biological storage includes carbon sequestration in plants, soils, or sediments of forests or wetlands, for example, which is also referred to as natural sink enhancement (NSE). Secondly, product storage involves incorporating carbon into carbon-based products like construction and insulation materials or biochar. Lastly, CO_2_ released during bioenergy provision can be captured and stored underground in geological formations. The bio-based lens offers a varied set of approaches that are largely already in use and well-developed.

Bio-based concepts offer multiple co-benefits beyond carbon removal, such as energy generation (electricity, heat, fuels in case of BECCS), construction materials production, or delivering ecosystem services (e.g., enhancing air and soil quality, protecting biodiversity). Moreover, many bio-based concepts require minimal investment and are maintenance-friendly, reducing costs^14–16^. By focusing on bio-based solutions, actions can be streamlined to achieve both carbon removal and a range of co-benefits, contributing to sustainable and climate-resilient practices. However, further implementation of bio-based CDR concepts comes along with several challenges such as competition for land and other resources, durability and reversal risk of CO_2_ storage, the impact of ongoing climate change on the stability of storing carbon in natural sinks, and other trade-offs. The challenges differ greatly between the concepts, and focussing on the different dynamics allows for a targeted comparison.

Hence, the variety of bio-based concepts calls for a systematic and FAIR (Findable, Accessible, Interoperable, and Reusable) data-driven knowledge collection to better understand their potential but also possible co-benefits and side effects. Users of such information may include decision makers and analysts, industrial actors (operators, research and development organisations), academia, non-governmental organisations, etc.^17^.

In this study, for the first time, an extensive and multi-dimensional assessment is made of bio-based CDR concepts in Germany. The following concept areas were investigated for this study: agriculture and soils, peatland rewetting and paludiculture, forest management practices, long-lived biomass-based building materials, and BECCS. A description of the concepts and specific research gaps is included in Annex 1. Generally, research gaps include site conditions and carbon accumulation rates for carbon sequestration in soils and above-ground biomass, scale-up and research into renewable building materials, including from paludiculture biomass, and pilot projects for novel CDR methods.

We intend to provide new insights into the dynamics of bio-based CDR in Germany. The focus on dynamics stems from the fact that bio-based CDR is embedded in highly variable systems with constantly interacting elements. For instance, the availability of resources influences the cost, the cost influences the scale of deployment, the scale influences the time needed for a specific carbon quantity to be captured and vice-versa. Investigating the dynamics allows us to reveal patterns inside the different system boundaries and identify possible points of intervention. Therefore, the investigated bio-based CDR solutions were compared with the aim of capturing the specific characteristics and differences between such diverse concepts.

## Methods

**Fig. 1:**
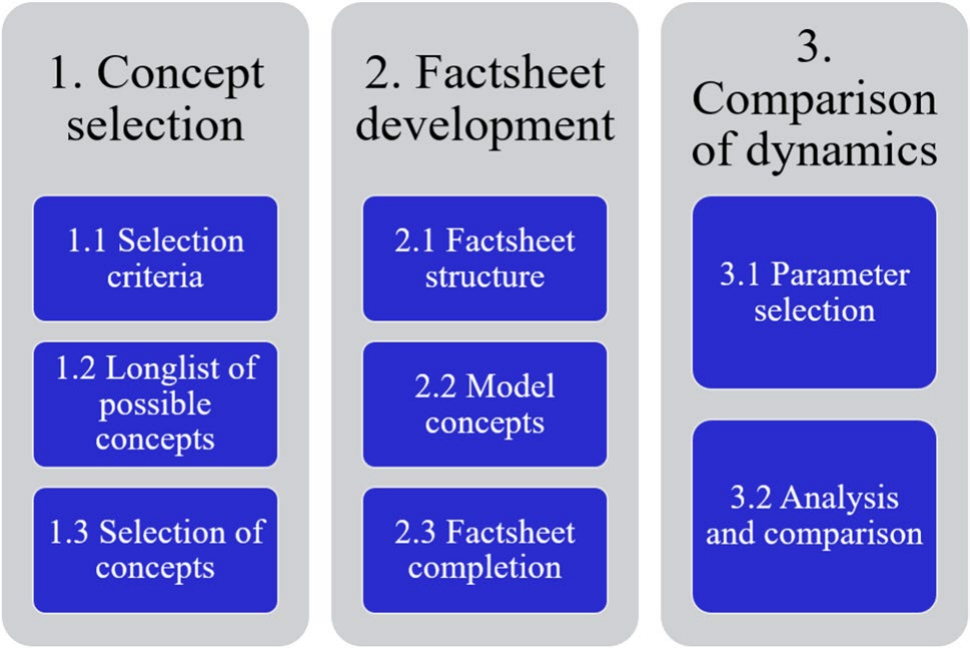
Methodological approach for concept selection, factsheet development and comparison of the concepts’ dynamics.

The results were generated in a three-step approach, which is summarised in Fig. 1 and further described in the following sub-sections.

### Concept selection

**Fig. 2:**
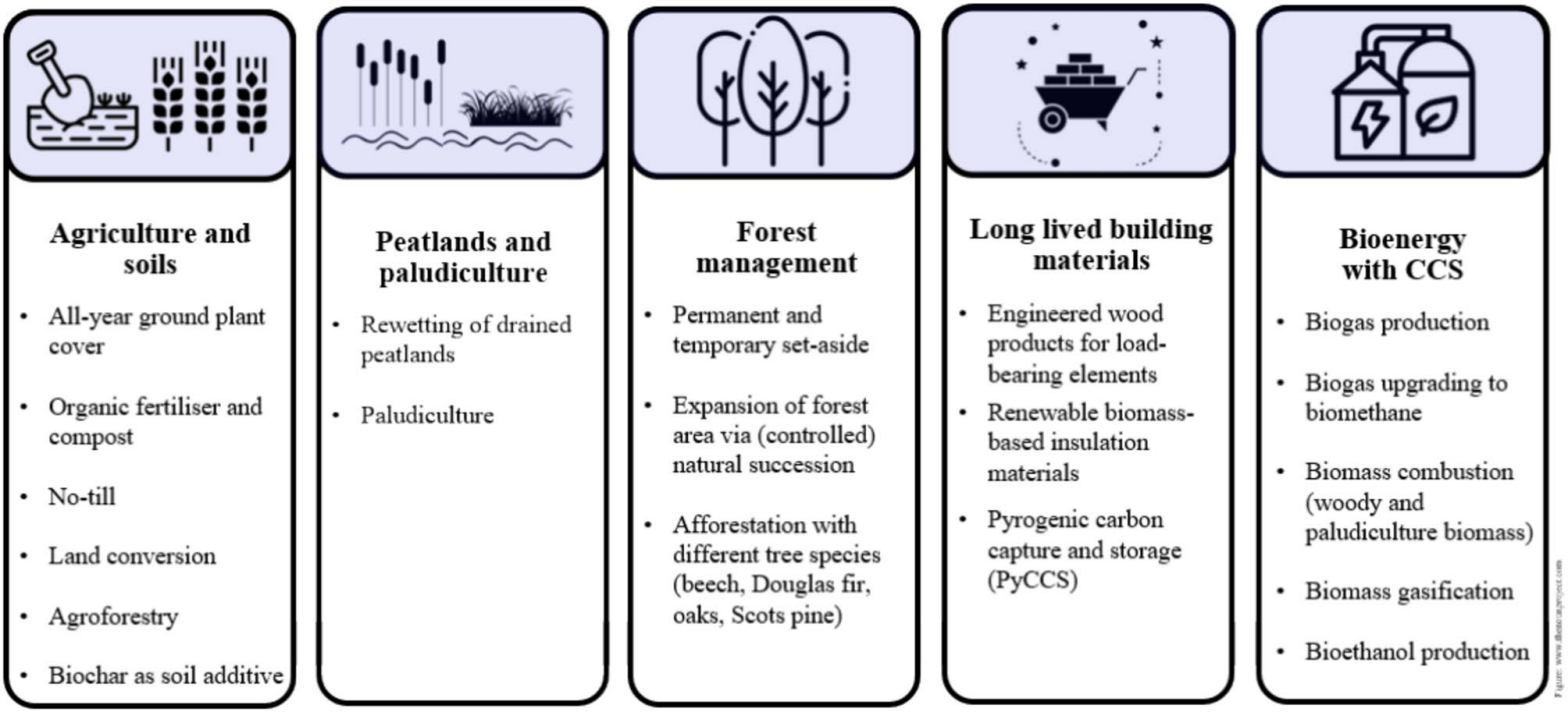
Overview over the bio-based CDR concepts chosen for the study.

To cover the wide range of possible biological CDR methods in a factsheets collection, we followed the selection criteria of (1) potential provision of net negative emissions when considering the entire life cycle, (2) potential long-term (> 100 years) carbon storage for the predominant mass fraction of the carbon extracted, and (3) use of biomass or biogenic CO_2_. We compiled a longlist of possible concepts meeting these criteria based on extensive literature research. In Annex 6, the longlist as well as the selection of concepts is presented. We selected 19 concepts from five areas (Fig. 2) applying three additional criteria: (1) the implementation in Germany is not impeded by geobiophysical conditions, (2) the implementation in Germany is possible on climate-relevant scales, (3) the technology readiness level is at least equal to 6, i.e., the technology is demonstrated in relevant environment^18^.

### Factsheet development

The factsheet structure was created based on the CDR technology assessment matrix by Ref.^19^. A representative example was developed for each concept to quantify CO_2_ removal potentials, costs, energy and material requirements, and outputs. The concepts were then assessed in technological, systemic, environmental, governance-related, economic, and social dimensions and described in the standardised factsheets using a total of 41 parameters. An overview over the entire set of parameters including their definitions is provided in Annex 2.

The data collection for the factsheets mainly relied on secondary data from the scientific and grey literature for the qualitative parameters, which was complemented by expert judgements. The quantitative data collection required setting temporal and spatial system boundaries. For the NSE concepts, the system boundary was set to 25 years, i.e., approximately the target date of the German Climate Protection Act, to account for (some of the) temporal removal dynamics. The data is shown as an average of these 25 years and is calculated per hectare, unless stated otherwise. For building materials, the hectare reference is assimilated by considering the construction of 6 multi-family and 6 single-family homes on one hectare. In the case of BECCS, the retrofitting of model plants is assumed, following the avoided costs methodology^20^. Therefore, the capture unit (if not already part of the model plant, as in the case of biomethane and bioethanol production), conditioning, transporting, and storing the gaseous CO_2_ lie within the system boundaries.

In some cases, where the system boundaries had to be interpreted cautiously due to missing or diverging literature values, divergence was made transparent.

The collected data and information is not only relevant for this project but for a broad range of further projects in the field of bioeconomy for stakeholders from both research and industry. Therefore, the data is made effective for diverse purposes by utilising the FAIR data principles for data management. To achieve this, the data (and its metadata) is published under an open licence on an openly accessible repository (see data availability statement). Furthermore, the data is post-processed for interactive browsing in a web application (https://datalab.dbfz.de/bionet) which utilises the same underlying data structure. Thereby, open standards for human and machine readability are met as the data is accessible as PDF fact sheets and as JSON files, both in long-term storage. By complying with the FAIR principles, scientific work and knowledge are improved and advanced, rendering it easier for everyone to (re-)use the data^21–23^.

### Comparison of dynamics

From the vast parameter database, the parameters that best reflect the diversity in dynamics were selected for analysing and comparing the bio-based CDR concepts. We show and discuss the dynamics in terms of (1) changes of CO_2_ removal potentials over time, and (2) variability of expenses. We display the CO_2_ removal dynamics graphically, and we provide overview tables with the CO_2_ removal values in Annex 3 as well as resource requirements for upscaling the concepts in Annex 4.

#### CO_2_ removal potentials

The values for CO_2_ removal potentials were taken from literature for the NSE concepts, and calculated based on literature data for the building materials and BECCS concepts. If the CO_2_ removal potentials were not available for the starting year in the literature, the averaged CO_2_ removal potential in the first 20 years, as stated in the factsheets, was used, except for the peatland concepts, where the first 15 years were chosen. To allow for comparison among the different concept areas, the graphics on temporal removal dynamics show relative rather than absolute CO_2_ removal values.

#### Input and output

The input and output data was taken from the literature. If own calculations were made, this was made transparent.

#### GHG emissions

In addition to CO_2_ removals, the greenhouse gas (GHG) emissions from deployment of the concepts were also analysed and reported in the factsheets. Apart from CO_2_, GHG emissions also include nitrous oxide and methane. Methane emissions play a major role in the rewetting of peatlands (see Annex 3). The values for the GHG emissions were taken from the literature. For the BECCS concept, the values are further processedstandard values from the Renewable Energy Directive^24^. All these values relate to the global warming potential of a greenhouse gas over a period of 100 years.

#### Costs

Based on the definition by Ref.^25^, the CAPEX (Capital Expenditures) include all longer-term investments (e.g. for construction, machinery, buildings, initial equipment, etc.) incl. expenditure for maintenance and repairs. The OPEX (Operating Expenditures) comprise all expenditures for the continuous assurance of a functioning operation of a concept, incl. expenditures for raw materials and operating materials, energy, staff and for administration, insurance, levies, distribution, etc. The CO_2_ removal expenses are calculated based on CAPEX and OPEX under the assumption of a fully established system.

## Results and discussion

The following section presents highlights from the comparison of temporal removal dynamics and expenses for bio-based CDR. In the supplementary information, a more extensive discussion on quantitative CO_2_ removal values (Annex 3), on the upscaling potential of the concepts to 1 million tons CO_2_ removed (Annex 4) and an outlook on the deployment of the concepts (Annex 5) provides important additions for the full understanding of the study.

### CO_2_ removal dynamics depend on the type of carbon sink

Temporal CO_2_ removal dynamics must be addressed when designing certification frameworks and crediting schemes, as laid out in the proposal for an EU certification of carbon removals^26^. Therefore, assessing the timing and storage durability of CO_2_ is crucial for bio-based CDR, especially due to the vulnerability of bio-based systems to environmental changes (e.g., Ref.^27^). The qualitative comparison of temporal CO_2_ removal changes highlights the differences in carbon sink dynamics among the concepts. The quantitative values of CO_2_ removal potentials for each concept are described and listed in Annex 3. Sudden deployment was assumed to allow for a comparison between carbon fluxes after implementation. If different deployment speeds were included, the common comparison basis would be lost.

#### Temporal removal dynamics for natural sink enhancement concepts

**Fig. 3:**
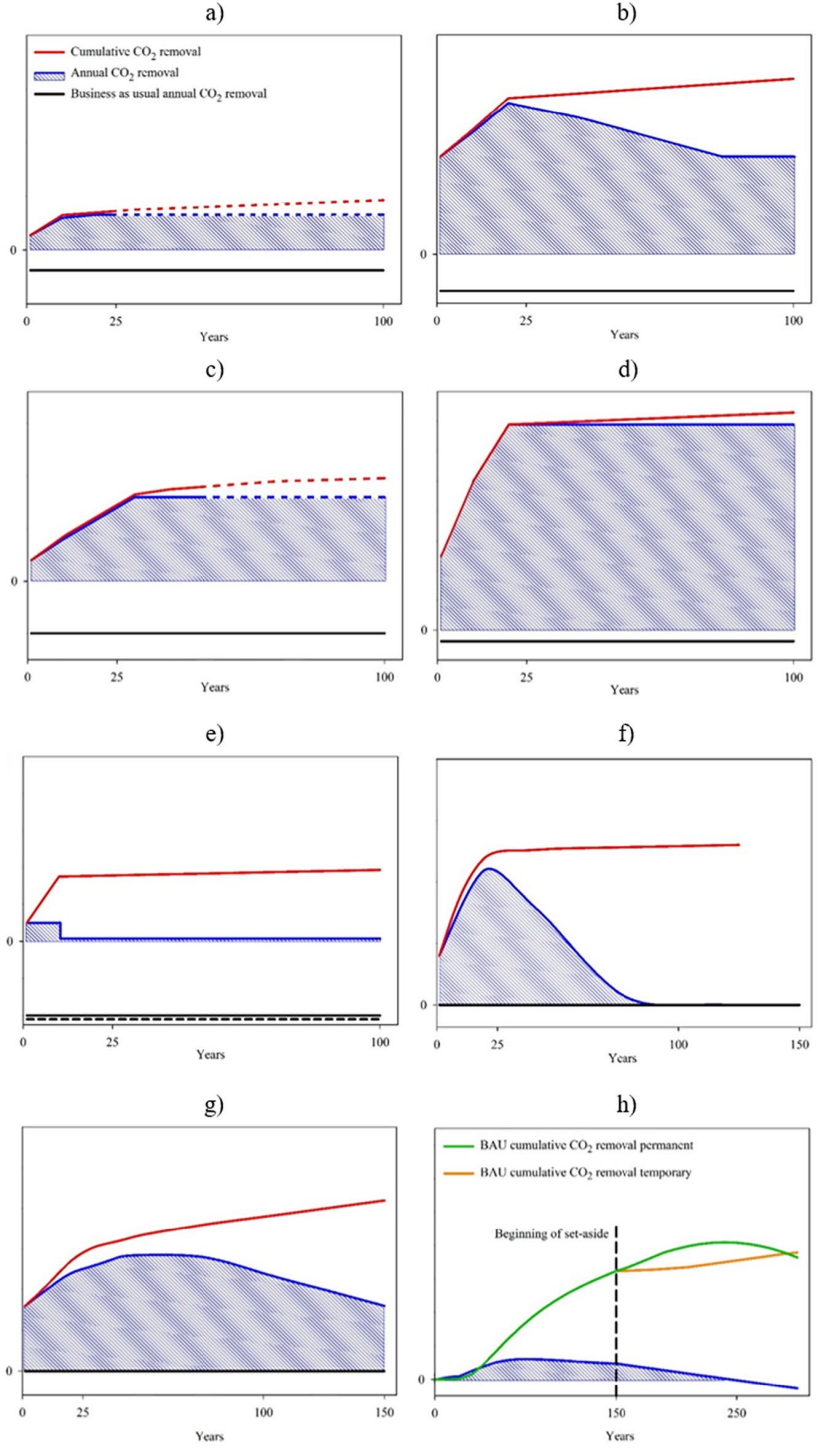
Conceptual (not quantitative) annual and cumulative CO_2_ removal dynamics of natural sink enhancement concepts over time, starting in year 1 after implementation assuming no gradual ramp-up. (**a**) all-year groundcover & no-till, (**b**) land conversion, (**c**) organic fertiliser/compost & biochar, (**d**) agroforestry, (**e**) peatland rewetting [due to lack of data, the annual removal is shown without gradual increase and decrease], (**f**) afforestation with pioneer trees, (**g**) afforestation with climax trees (both (**f**) and (**g**) under forest management but without final felling), (**h**) set-aside of old (beech) stands. The business-as-usual (BAU) is conventional agriculture (**a**–**g**) and beech forestry without the CDR measure (**h**). For (**a**–**g**), black indicates BAU, blue the annual removal and red the cumulative removal. For h, green indicates BAU cumulative CO_2_ removal for permanent set-aside and orange indicates BAU cumulative CO_2_ removal for temporary set-aside. BAU for (**e**) peatland rewetting with solid line indicates grassland on drained organic soil and dashed line arable land on drained organic soil.

For the natural sink enhancement concepts related to mainly sequestering carbon in the soil (i.e., peatland rewetting and agriculture & soil concepts), the main removal effect occurs in the first years to decades of implementing the concept (see Fig. 3a–e for schematic, i.e., non-quantitative illustrations). After that, the additional removal is strongly reduced and for some systems a saturation can be reached. In peatlands, short-term changes result in considerable removal in the first years after rewetting^28^, whereas long-term peat accumulation is much slower^29^. Depending on the management type of mineral soils, there is even a risk of reversal, e.g., due to ploughing, turning a carbon sink into a source (e.g., Ref.^30^). In turn, the C storage in rewetted peatlands is permanent if they remain wet in the future (Fig. 3e). External events like wildfires, droughts or floods can also lead to the re-release of greenhouse gases. For this reason, the graphs of the annual as well as the cumulative CO_2_ removal potential are shown dashed at the end (Fig. 3a,c). Note that if no rewetting takes place, CO_2_ is emitted from the drained peatland, which is why the business-as-usual case (Fig. 3e) is displayed with a negative CO_2_ removal (i.e., positive CO_2_ emissions). Upon rewetting, methane emissions occur. Methane is a short-lived, but strong greenhouse gas^13^. Even though a carbon sink can be established, it will take time for the rewetted peatland to become cooling. Before cooling sets in, rewetting will effectively reduce the warming effect of peatlands^31,32^.

When a major storage effect happens in the aboveground biomass and not only in the soil (i.e., in forest management, agroforestry, or conversion from cropland to permanent grassland), the annual removal is lower in the first years and then increases, depending on biomass growth rates. The additional yearly uptake reaches a maximum before the saturation stage, in which the same factors as above may lead to the re-release of greenhouse gases.

Figure 3f,g,h illustrates the forestry concepts, again schematically. The development of CO_2_ removal within a forest stand—even a managed one—is not linear over time, as it depends on the age-dependent dynamics of tree growth (see e.g. the yield tables compiled by Schober^33^, which show the development of forest stands under standardised forest management conditions). For afforestation, a differentiation was made between the CO_2_ removal potential of early successional (“pioneer”) tree species like e.g. birch (Fig. 3f) and late successional (“climax”) tree species like e.g. beech (Fig. 3g), with pioneer species providing most of their CO_2_ removal in the first decades, while climax species reach their maximum removal potential later, even if their overall storage capacity ultimately is higher. Note that the final felling is not shown in Fig. 3, as in practice its timing is highly dependent on growing conditions and management objectives and may extend over longer periods of time. In the case of the set-aside of old beech stands from an age of 100 years (Fig. 3h), business-as-usual corresponds to a continuation of the management of the old beech forest. Permanent set-aside initially has a positive effect on the annual CO_2_ removal potential. However, after a certain time since abandonment, the culmination is reached as the additional growth potential is exhausted, the removal potential decreases, and beginning decay can also reduce the carbon stocks accumulated until that time^34^. In the case of temporary set-aside over a period of 25 years, for example, the further development will depend on whether and in what way management is resumed. Due to the natural mortality and limited lifespan of trees, the additional annual CO_2_ removal approaches zero in all cases after several decades or even centuries.

#### Temporal removal dynamics for technical sink concepts

**Fig. 4:**
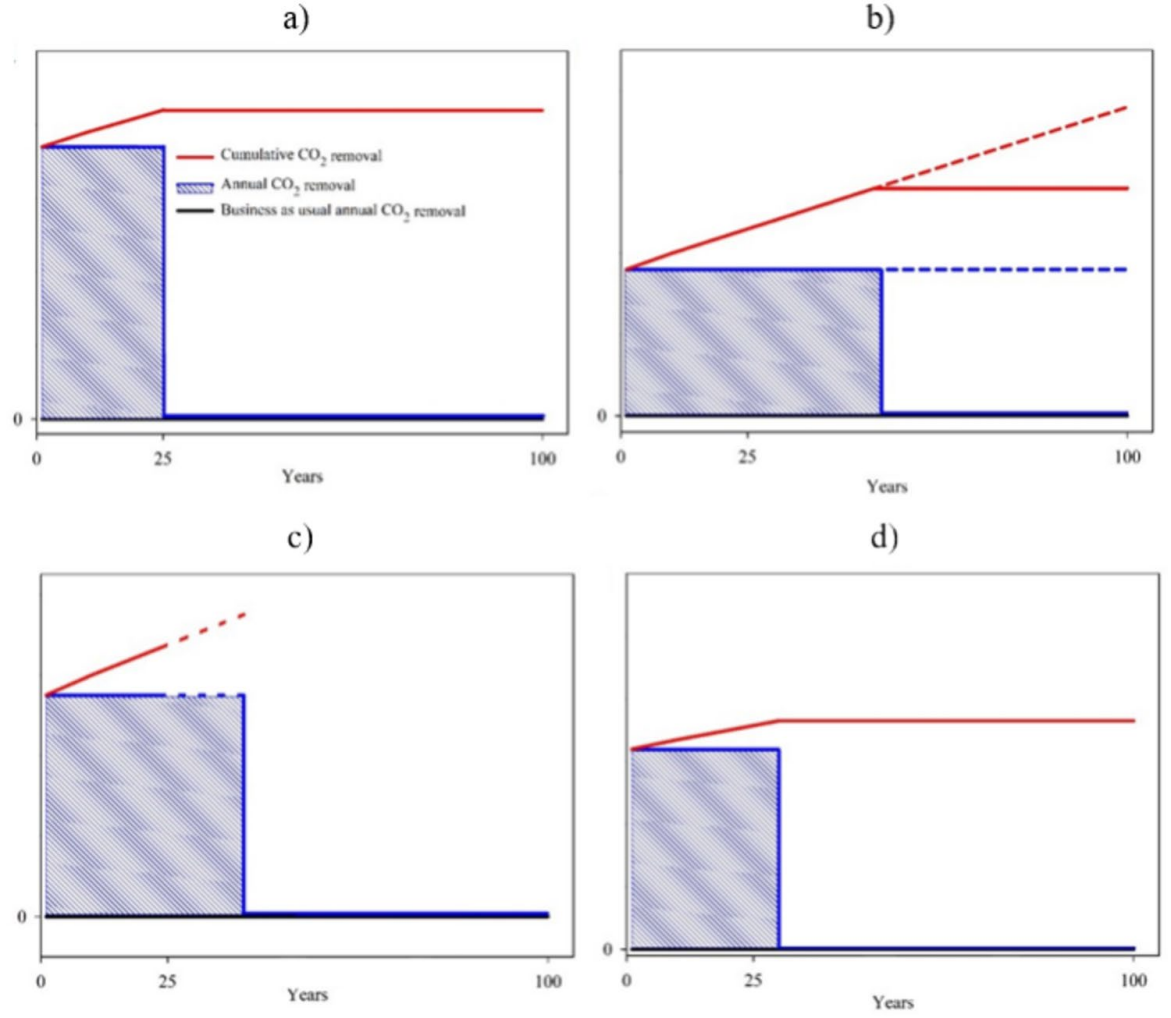
Conceptual (not quantitative) annual and cumulative CO_2_ removal dynamics of biomass-based building materials and BECCS over time, starting in year 1 of deployment assuming no gradual ramp-up. (**a**) Insulation materials, (**b**) wood-based buildings, (**c**) PyCCS, (**d**) BECCS. BAU is non-bio-based building materials (**a**–**c**) and plant operation without a CO_2_ capture unit (**d**). For all figures, black indicates the BAU, blue the annual removal and red the cumulative removal.

Storing biogenic carbon in the built environment via biomass-based CO_2_-negative building materials faces building stock-related rather than biophysical constraints. For instance, the building stock is assumed to be fully energetically renovated after 25 years in line with the national energetic renovation goals until 2050^35^ (schematically illustrated in Fig. 4a). The dotted lines for wood-based building constructions (Fig. 4b) indicate the physically possible additional removal through further construction.

For the considered timeframe of implementation (50 years for wood construction, 25 years for energetic renovation and 25–40 years for biochar use), annually constant rates of building (which removes around 2120 t CO_2_ ha^−1^ a^−1^), renovation, and replacement are assumed. After the time of implementation, the cumulative removal remains constant, assuming no changes in the built environment that lead to net CO_2_ emissions back into the atmosphere.

In the case of PyCCS (Fig. 4c), urban sealed areas are replaced with biochar-based materials such as pavement materials, unsealing with French drain substrates and additional green roof capacities for increasing urban water holding capacities. Because of the multitude of applications, the annual removal depends on the capacity of the pyrolysis plants for biochar production for which an operating technical life time of 25 years is assumed with possible extension to 40 years (indicated by dotted lines,^36,37^).

The storage of CO_2_ in underground reservoirs can be seen as permanent^38^, turning BECCS into an option that durably extracts CO_2_ from the atmosphere. The efficiency of CO_2_ capture systems in BECCS plants remains rather stable over the years of plant operation^39^, providing a steady stream of CO_2_. However, fluctuations in annual removal rates may still occur, e.g., in reaction to changes in biomass availability and flexible plant operation. In this regard a base load plant operation is favourable for a greater stability of the system. For displaying the removal dynamics schematically (Fig. 4d), the operation of a single plant with a lifetime of 30 years is assumed. While the dynamics are similar for different bioenergy plants, the absolute values for the annual CO_2_ removal potential vary greatly depending on the plant size.

In conclusion, CO_2_ removal potential values of CDR concepts vary depending on the timeframe examined. When comparing concepts that operate in different systems, the temporal system boundaries must be chosen with care to avoid false conclusions. The storage durability should then be assessed in connection with temporal dynamics and removal potentials^27^. Here, we focussed on the first 25 years of implementation to stay consistent with the 2045 horizon set by the German Climate Protection Act^4^. In order not to encourage misconceptions, however, we also show the longer-term dynamics that are induced by deploying the CDR concepts in a timeframe of 100–200 years. Another limitation is the speed of deployment: while the figures in this section are based on the assumption of sudden deployment, in reality ramp-up effects may play an important role in limiting the CO_2_ removal potential.

### Expenses are highly variable among the concepts

In general, CO_2_ removal expenses are low for most of the forest- and agriculture-based concepts compared to the expenses associated with CO_2_ removal through building materials and BECCS. The heterogeneity of reference systems and units complicates a direct comparison, especially when viewing the expenses in the context of additional factors such as reliability of permanent CO_2_ storage. Also, the consideration of additionality has a high influence on expenses, but its quantification is highly context-specific. For instance, on the one hand, land owners likely lose income through afforestation on their land^14^. On the other hand, building with renewable bio-based materials can be cheaper than the conventional material alternative, depending on regional prices^40^. The CO_2_ removal expenses and the CAPEX and OPEX values presented in this chapter partly show wide ranges for individual concepts, because expenses are highly context-dependent and it is difficult to give a general estimate.

#### Agriculture and soils

Depending on the agricultural concept applied, CO_2_ removal expenses range between 20 and 30 € t CO_2_^−1^ ha^−1^ (organic fertiliser and compost, biochar and all-year ground cover) or between 50 and 80 € t CO_2_^−1^ ha^−1^ (no-till and land conversion). The highest CO_2_ removal expenses are found in the agroforestry concept, where they can range from 125 to 520 € t CO_2_^−1^ ha^−1^^41,42^. On average, the CAPEX of the concepts can amount to 156,000 €, while it is lowest in the no-till concept (70,000 €). The OPEX for a farm with an average agricultural area of 63 ha can range between 70 and 500 € ha^−1^, depending on the concept applied, with maximum expenses of 819 € ha^−1^when biochar is applied^43^. The expenses can fluctuate greatly, especially due to future price adjustments or price increases.

#### Peatland rewetting and paludiculture

The expenses for CO_2_ removal in peatlands and paludiculture cannot be determined due to missing data. The expenses of a GHG emission reduction (abatement costs) resulting from Moorfutures projects was 35–67 € t CO_2_eq^−1^^44^.

The expenses for rewetting largely consist of one-time payments. The values are in the range of 1065–17,555 € ha^−1^^45^. Expenses for securing the land could cause extra expenses of 1500–18,150 € ha^−1^ (Ref.^44^, for 2022). Every site has different preconditions, which is also shown by the wide range in the expenses. The reference projects primarily had nature conservation goals. Rewetting projects for CDR may be more expensive (e.g., an active irrigation system for stably high water levels). The expenses also vary greatly depending on the effort required for the technical implementation (nature conservation requirements, impact on settlements and infrastructure) and to a lesser extent for planning and approval. For larger areas, effects of scale may occur (i.e., expenses may decrease from small to larger implementation projects).

In addition to the initial expenses, follow-up expenses in the form of area-based charges, care and maintenance expenses must also be calculated for rewetting projects, especially if they are accompanied by land acquisition or abandonment of use. They amount to approx. 95–625 € ha^−1^ a^−1^^45^. For monitoring, 10% of the construction and planning expenses are usually set^44^, but expenses can be significantly higher, e.g., 810–1040 € ha^−1^ a^−1^ for permanent active water management^46^.

#### Forest management practices

The range of CO_2_ removal expenses for afforestation (without considering land acquisition costs and costs of crop protection) is between < 10 € t CO_2_^−1^ and > 100 € t CO_2_^−1^ for the tree species beech, Douglas fir, oak, and pine, respectively^47^. According to the same source the investment expenses for comprehensive afforestation with the use of machinery and without the preparation of soils (e.g. personnel expenses, purchase of plant material and seeds, maintenance, planting expenses) are about 1600–5800 € ha^−1^, depending on the tree species. More recent data from^48^ lie within the same range. Operating expenses include expenses of crop protection and amount to approx. 1100–1800 € ha^−1^^47^. A modification of afforestation is the natural succession. There are no direct investment expenditures, but the establishment of stocks is more uncertain and also usually delayed. Setting aside old beech forests does not cause investment and operating expenses, apart from possibly increased expenses for traffic safety. However, temporary set-aside implies interest costs for the forest owner and additional calamity risks due to delayed harvesting; beyond this, the stands may lose value. Permanent set-aside even implies much higher costs, because the forest owner then loses all capital accumulated in the forest stands (these are roughly between 20,000 and 30,000 € ha^−1^ for beech, depending on accumulated timber volume, wood quality, and timber prices;^49,50^). All data quoted here relate to the individual circumstances of the respective case study. Therefore, these figures are only indicative.

#### Long-lived biomass-based building materials

Specific expenses for carbon storage in buildings are generally not accounted for in conventional building projects, therefore the focus will be directed to opportunity costs and additionality criteria. Expenses of projects for constructing new buildings and for energetic renovation differ significantly between German regions^40^. Therefore, the expenses associated with using bio-based materials account for a lower fraction of expenses in regions where expenses are high and vice-versa. Generally, in southern regions such as Bavaria and Baden-Württemberg expenses for construction and of energetic renovation are higher and in northern and eastern regions expenses are lower.

For PyCCS in the built environment, the CO_2_ removal expenses are considered to range around 150 € t CO_2_^−1^. The expenses for construction of e.g. infiltration troughs or green roof substrates are considered to be either sunk costs when only substrates are replaced, or independent expenses which are associated to the primary service the measures intend to deliver (aside of negative emissions), i.e., stormwater infiltration and storage, urban cooling or traffic space provisioning.

The deployment of insulation materials for energetic renovation is normally optimised for only contributing to buildings energy efficiency and for energy expenses savings in the long-term, therefore optimisation conflicts considering cost efficiency and the amount of removed CO_2_ need to be accounted for. Materials with higher thermal transmittance and higher thickness might reach higher carbon storage but also higher expenses as the material demand is increased. For insulation materials, the removal expenses range from 0 to 300 € t CO_2_^−1^ depending on the regional opportunity costs^40^.

#### Bioenergy with carbon capture and storage

In the case of BECCS, the calculation of expenses follows the avoided cost methodology by Ref.^20^, considering the expenses associated with capturing, transporting and storing a ton of CO_2_ instead of releasing it to the atmosphere. Therefore, the CAPEX of retrofitting existing bioenergy plants was found to be in the range of 60,000 to 4.5 million € a^−1^, while the CCS-associated OPEX was between 100,000 and 18.6 million € a^−1^ per bioenergy plant (the min–max values are for heat plants fuelled by paludi biomass and for bioethanol plants, respectively). Due to the close relation between plant size and CO_2_ removal potential, the comparability of expenses is more straightforward for CO_2_ removal expenses. These are in more similar ranges for the model plants, amounting to about 83 € t CO_2_^−1^ for biomethane and bioethanol production, and to about 139 € t CO_2_^−1^ for the BECCS plants with post-combustion capture (own calculation based on Ref.^51^). The lower expenses for the biomethane and bioethanol model plants stem from the high purity of the CO_2_ stream produced endogenously. In contrast, capturing CO_2_ from a flue gas stream with a concentration of about 10–15% CO_2_ increases expenses^52^. The calculated values are within the ranges given in the literature for different BECCS processes^25,53,54^, which vary widely due to the multitude of cost influencing factors, including infrastructure availability for transport and storage, storage distances, and feedstock prices. Further assessments, which also compare BECCS expenses to fossil-based CCS, are needed^55^.

When comparing dynamics of expenses across the different CDR concepts, there are many concepts for which they are expected to decrease over time due to learning curve effects, especially for bio-based building materials and BECCS^17^. However, this cannot be generalised for all concepts. In rewetting projects, for instance, the areas considered low-hanging fruits are expected to be rewetted first, leading to higher rewetting expenses for the harder-to-access areas.

## Conclusions and outlook

The developed factsheets are suitable for comparing bio-based CDR concepts because a wide portfolio of the concepts with the highest potential was selected. We provide a sound basis for comparability which aims to cover many aspects from economics, resource base and environmental impacts to social and political implications. Our analysis goes beyond simply examining the CDR effect, and beyond the status quo by incorporating long-term potentials.

Uncertainties and gaps arise from difficulties in acquiring data, especially economic data. The concept selection, the choice of system boundaries, and the assumptions made for calculations are only one alternative of describing the concepts, which limits the generalisation of results. Inconsistencies in literature data assumptions from different sources further complicate comparability. The data thus allows for giving a general idea about potentials and for discussing tendencies of the different concepts.

Further research should focus on investigating possible interactions between concepts by systematically modelling resource flows to show distribution and competition patterns. Moreover, the regionally different preconditions should be mapped in more detail. Derived from the potentials and limitations of this study, the factsheets and the investigation of dynamics is a strong basis for modelling the future contribution of the concepts to the German climate neutrality target. The data collection is also useful for stakeholder engagement to discuss regional issues.

Table 1 provides a summary of the main findings from analysing the temporal and expenses dynamics for the German context.

**Table 1:** Summarized main results from the analysis of temporal and expenses dynamics for Germany.

Parameter	Concept area	Result
Temporal dynamics of CO_2_ removal rates	Natural sink enhancement	Gradually increasing rates, followed by a saturation and potentially a decrease after few decades. The time horizons depend on plant growth periods: forest-based concepts have the longest time horizons due to longer vegetation cycles than in agriculture and peatlands. Higher uncertainty than technical sinks regarding storage durability
	Long-lived biomass materials	Annually constant removal rates are assumed during construction phase, additional removal drops to zero afterwards. Lower uncertainty than natural sinks regarding storage durability
	BECCS	Annually constant removal rates are assumed during operation which drop to zero afterwards. Lower uncertainty than natural sinks regarding storage durability
Expenses for removing 1 t CO_2_ from the atmosphere	Natural sink enhancement	Generally well below 100 €, where data was available Minimum: 8 € t CO_2_^−1^ for afforestation with Douglas fir. Maximum: 520 € t CO_2_^−1^ for agroforestry
	Long-lived biomass materials	Large uncertainties due to differences in regional prices Minimum: 20 € t CO_2_^−1^. Maximum: 560 € t CO_2_^−1^
	BECCS	Expenses calculated for capture unit add-on to existing bioenergy plants. Minimum: 81 € t CO_2_^−1^. Maximum: 139 € t CO_2_^−1^(add-on to existing bioenergy plants)

In conclusion, when tapping into these CDR potentials, the methods' intertwined synergies and trade-offs with the transforming energy systems, with land use change dynamics and with climate change dynamics need to be assessed. Concepts with relatively low CO_2_ removal potential and/or low reliability of storage durability on one hand may compensate by delivering crucial biodiversity functions on the other hand. Concepts with high investment costs due to a still lacking CO_2_ transport and storage infrastructure could in turn have a constant and high CO_2_ removal.

When looking at the current deployment of bio-based CDR concepts, there are best-practice projects in place for all examined concepts. Examples include agricultural measures like agroforestry systems or biochar as a soil additive, the rewetting of drained peatland and cultivating those areas with paludiculture, the conversion of forestry systems with more adapted species and more extensive forest cultivation, carbon storage in building materials like wood or straw for temporary storage and subsequent use for energy combustion, as well as capture of CO_2_ from various combustion processes or from biogas for storage in geological formations. Incentive instruments for many concepts promote the implementation through financial or structural support, but often not explicitly with the goal of CO_2_ removal. The natural sink enhancement concepts and some long-lived materials could be deployed immediately, whilst BECCS and ramping up construction materials still face legal and political barriers. Recommendations for the deployment of the individual bio-based CDR concepts are provided in Annex 5.

When moving from current implementation levels to exploring the full potential, these co-benefits should be taken into account. This can be best achieved in a holistic, portfolio-based approach to bio-based CDR which recognises the complex system dynamics to develop solutions. For many cases these integrated assessments, however, are still in their infancy of full systems understanding. The factsheets and the investigation of their different dynamics serve as a basis for these assessments. Thus, the primary motivation for CDR in the coming decades is its contribution to compensating for budget overshoot and residual emissions. Considering its long-term role past the current century, CDR methods are here to stay in order to increasingly contribute to planetary stewardship in the sense of stabilising the global climate trajectories once they have reached their individual scale of climate effectiveness.

### Supplementary Information

Supplementary Information.

## Data Availability

The factsheets generated and analysed during the current study are available in the OpenAgrar repository in German and English, 10.48480/x293-8050 (English) and 10.48480/tga8-t109 (German).
