# Supplementary material for: Dynamics of bio-based carbon dioxide removal in Germany
Source: Sci Rep. 2024 Sep 2;14:20395. doi: 10.1038/s41598-024-71017-x (PMC11369247; doi:10.1038/s41598-024-71017-x)
Supplement: Supplementary file 1 — Supplementary Information. [file 41598_2024_71017_MOESM1_ESM.docx]

Dynamics of bio-based carbon dioxide removal in Germany

**Annex 1: Concept descriptions and research gaps**

**Annex 2: Assessment framework definitions**

**Annex 3: CO_2_ removal data**

**Annex 4: Upscaling potential of the concepts to 1 million tons CO_2_ removed**

**Annex 5: Outlook on the deployment of the concepts**

**Annex 6: Longlist of bio-based CDR concepts and application of concept selection criteria**

Annex 1: Concept descriptions and research gaps

**Agriculture and soils.** There are different ways of managing farmland in order to increase the soil’s ability to absorb and sequester more carbon. Various studies have reported an increase in organic carbon in the soil under soil carbon sequestration methods [1]. Nevertheless, the sequestration rates varied greatly, as the studies took place under different conditions (e.g. soil properties, climatic conditions, farming method, crop grown) and assessed different timeframes. Furthermore, depending on the duration, some studies showed that some methods have little to no detectable effect on soil carbon accumulation, e.g., no-till [2], or that there is a need for further research to make reliable statements on the effectiveness of the respective method, e.g., biochar application to soils. With increased C input and reduced decomposition rates, soil C stocks tend to reach a new equilibrium, whereby soil saturation can be achieved, resulting in a decrease in additional soil C input over the years [3, 4]. Management practices designed to accumulate carbon in the soil must be maintained over the long term due to a reversal risk, i.e., a carbon sink becoming a source again.

**Peatland rewetting and paludiculture.** Peatland rewetting, i.e., restoring a stable water table at the ground surface, is known since the 1970s from nature conservation for bogs and fens [5, 6]. In Germany, over 1 million ha of peatlands are currently drained and could potentially be rewetted. Rewetting can immediately reduce or stop net C loss and can also lead to new C sequestration in the litter layer and peat. Assumptions on the CDR potential come from derivation from natural peatlands where C stocks have been built up over thousands of years, or from rewetting projects or paludiculture pilot sites. The water level and dynamics are the key factors influencing GHG emissions in peatlands [7]. There are still research gaps on the effects of site conditions, the type of vegetation or the management through water and farming practices. A rewetting project’s design and costs may vary depending on whether the main goal is C sequestration or nature conservation. An overall area-specific determination of the potential and scaling for Germany is difficult because of heterogeneous site conditions and land use conflicts, and has not been done yet. The combination of rewetting with paludiculture, i.e., the productive land use on wet and rewetted peatlands, could increase the CDR potential through annual biomass harvest and use for BECCS or long-lived products [8]. But many research questions are still open. For example, a complete life cycle assessment with paludiculture biomass-based products has not been done yet for Germany.

**Forest management practices.** Forest management, i.e. establishing and/or manipulating forest ecosystems, is a traditional measure that has been researched and optimised since the end of the fifteenth century [9]. There are various short-term and long-term forest-related measures that enable C sequestration [10], including changing management practices in the forest sector (which can also favour an increased use of woody biomass in other sectors). In new afforestations, a considerable amount of carbon, depending on the tree species, can be sequestered in the first two decades [11], thus building up a carbon storage that lasts for several decades or even centuries [12]. A cost-effective alternative is to allow natural succession, although this may delay the establishment of the tree stands. Since tree growth (and thus sequestration) usually reaches its maximum only after the first two decades, both afforestation and succession are measures that are even more effective in the long term. In already existing forest stands, both increment and wood stocks can be increased to a limited extent through forestry measures. One of these is the temporary or permanent abandonment of wood harvesting, which is particularly suitable for beech stands due to the growth dynamics of this tree species [13].

**Long-lived biomass-based building materials.** Incorporating biomass-based building materials into the material stocks of the building sector is a CDR measure which also contributes to GHG abatement [14–17]. Research is intensifying on the global scale as well as national and regional scales. The German production capacities for engineered wood products and for biomass-based insulation materials are individually part of a growing niche segment, whereas the production of biochar substrates in pyrolysis plants is still in the early ramp-up of market-relevant quantities to cover growing future demands of biochar carbon removal (BCR) systems. In particular, region-specific scale-up factors and impediments as well as regionalised additionality criteria and benchmarks are often underexplored. Furthermore, literature and studies on future resource competition when different CDR technologies are scaled up simultaneously are still lacking.

**Bioenergy with carbon capture and storage**. Bioenergy with carbon capture and storage (BECCS) is a CDR option that combines the utilisation of biomass (e.g., agricultural residues, waste, forest biomass, or energy crops) for energy generation (heat, electricity, fuels) with the capture and underground storage of CO_2_. There are many bioenergy technologies available to be used for BECCS (e.g., combustion, gasification, pyrolysis, anaerobic digestion, and fermentation), and three main processes of CO_2_ capture from flue gas or syngas (pre-, post- and oxy-combustion). BECCS technologies handle specific types of biomass, have specific resource needs, and operate on different scales. All of these elements add up to an extensive range of choices and decisions when it comes to configuring efficient BECCS systems. BECCS has been studied extensively since 2005 [18], with research focused on various aspects, e.g., techno-economic feasibility and system configurations [19–21], BECCS in socio-political contexts [22–25], or BECCS in scenario literature [26–29]. However, despite the progress made, several challenges and research gaps still exist. These mostly concern the costs, land and water use issues, upscaling feasibility, sustainability of biomass resources, energy efficiency, ability to deliver negative emissions, and lack of policy support or limited public acceptance [30, 31]. Additionally, successful BECCS deployment also depends on the availability of infrastructure for CCS, which still needs to be established in Germany. Up to date, there are only a few large BECCS power plants being planned but none are in operation or construction. The only large-scale BECCS facility with a capture capacity of 1.5 Mt CO_2_ per year in operation absorbs CO_2_ from ethanol production in the United States [32]. Undoubtedly, further research and testing different technologies on the pilot scale are needed to better understand technical and non-technical constraints and unlock the full potential of BECCS as a critical component of a low-carbon future.

Annex 2: Assessment framework definitions

**Table A.1** Categories, parameter names and parameter definitions of the assessment framework used to analyse the bio-based CDR concepts

| **Category** | **Parameter name** | **Parameter definition** |
| --- | --- | --- |
| Basic concept characteristics | Biophysical and socio-geographical conditions | Describes the necessary biophysical (e.g., soil condition, precipitation, temperature, solar radiation, etc.), and socio-geographical conditions of a concept (e.g., access to the sea, rural areas, availability of certain biomass, etc.). |
| Basic concept characteristics | Infrastructure | Describes the necessary infrastructure for a concept application, e.g., power grid, gas grid, CO_2_ grid, other transportation pathways, machinery, industrial plants, water supply/drainage, etc. |
| Basic concept characteristics | Maturity level | Describes the degree of maturity or development of a concept between the initial idea and a market-ready concept, which can be widely deployed. If applicable: Technology Readiness Level (scale 1-9). |
| Systemic parameters | Long term CO_2_ removal potential | Describes the net CO_2_ emissions removed by a concept (with assumption of permanent storage) in 2045, 2100, 2120. |
| Systemic parameters | Cumulative CO_2_ removal potential over 25 years | Estimates the minimum and maximum rate (range) at which CO_2_ is removed between 2020 and 2045 and beyond to 2120. |
| Systemic parameters | Permanence | Describes in what form (gaseous, liquid, solid) the CO_2_ is stored and where (in what kind of carbon reservoir, i.e., geological, terrestrial or marine biomass, terrestrial or marine soils/sediments, construction). Includes the expected length of storage and leakage risks. |
| Systemic parameters | Verifiability | Describes the ability to confirm the amount of CO_2_ removed, including the options to measure or estimate fluxes, carbon stock changes or leakages. |
| Systemic parameters | Combination options with other concepts | Examines what combinations with other concepts are worthwhile (e.g., because of higher resource efficiency, substitution effects, etc.). |
| Systemic parameters | Impacts from climate change | Determines whether the concept is influenced by climate change (positively or negatively). |
| Systemic parameters | Co-benefits from a systemic perspective | Describes benefits of the concept from a system integration perspective in addition to providing negative emissions, e.g. flexibility, modularity. |
| Input | Space requirement | Determines how much area is necessary for the concept deployment and what kind of area is required (agricultural area, forest area, urban area, marine area, etc.). |
| Input | Raw material demand | Determines whether any raw materials (e.g., biomass, minerals, auxiliary and operating materials, forestry regeneration material) are required to run the concept, including what kind and in what amount. |
| Input | Material flow redirection from other use paths | Describes whether and how the raw materials required for the use of the concept are already tied up in other utilisation paths (e.g., high value/low value uses). |
| Input | Energy demand | Determines and quantifies the necessary energy supply for the concept application. |
| Input | Labor | Determines how many jobs are required to implement and to run the concept, including bottlenecks or need for re-training. |
| Output | CO_2_ removal potential | Estimates the current expected net CO_2_ removal per year by the concept (under the assumption that the system is fully established, averaging over 20 years). |
| Output | GHG emissions | Estimates the net GHG emissions: GHG emissions from the background system, plus direct non-CO_2_ emissions, plus or minus net CO_2_ removed. |
| Output | GHG avoidance potential | Estimates net GHG avoidance through different cultivation and substitution of processes, energy carriers, materials compared to a reference system. |
| Output | Material products | Lists the material products generated by the concept. |
| Output | Energy output | Estimates the energy generated by the concept (electricity, heat, fuels). |
| Environmental parameters | Impacts on the water balance | Describes potential impacts on the water balance through the use of the concept, its water consumption and waste water quantities. |
| Environmental parameters | Soils | Describes substances released into the soil and changes of soil physical state (positive and negative). |
| Environmental parameters | Water | Describes substances released into the ground water, runoff water, sea water, and retention of nutrients. |
| Environmental parameters | Air | Describes substances released into the air incl. changes of air temperature (positive and negative impacts). |
| Environmental parameters | Noise | Describes the generation of noise (which can impact air, water, soils). |
| Environmental parameters | Impacts on flora and fauna | Describes what conservation or beneficial effects can result from the use of the concept (e.g., conservation and promotion of biodiversity, species protection for animals and plants, habitat creation), and what negative effects there are. |
| Environmental parameters | Contribution to improving further environmental quality goals | Describes which additional positive effects can be drawn from the concept besides negative emissions (e.g., local heat mitigation, reduced soil drought, flood control). |
| Institutional parameters | Regulatory framework | Describes permission requirements and regulations that must be complied with for implementation (e.g., BImschV, Federal Forest Act). |
| Institutional parameters | Further governance framework | Determines whether there are additional frameworks that currently support or obstruct the implementation of the concept (narrow, focused on single concepts, no general frameworks). |
| Economic parameters | CO_2_ removal costs | Estimates the CO_2_ removal cost under the assumption that the system is fully established (incl. consideration of opportunity costs). Calculated from CAPEX (annualized), OPEX and CO_2_ removal potential. |
| Economic parameters | CAPEX (Capital Expenditures) | Estimates all longer-term investments or capital expenditure for the concept (e.g. for construction, machinery, land, buildings, initial equipment) incl. expenditure for maintenance and repairs. |
| Economic parameters | OPEX (Operational Expenditures) | Estimates all expenditures for the continuous assurance of a functioning operation of a concept, incl. expenditures for raw materials and operating materials, energy, staff, and for administration, insurance, levies, distribution, etc. |
| Economic parameters | Further economic impacts | Describes whether there is additional economic value generated, e.g., by increasing wages, taxes, land values, effects by attracting labour, as well as negative economic impacts of the concept. |
| Economic parameters | Fair conditions of competition | Describes whether there are distortions of competition as a result of different levels of subsidies and to what extent the carbon price and/or other externalities are priced into the respective market environment of the concept. |
| Social parameters | Social perceptions and assessments | Describes whether there is any strong expected advocacy/resistance of the concept by the local population, the economy and socio-political key actors (at present). |
| Social parameters | Risk assessment | Describes the stakeholder assessment of the risks associated with the operation and location of a concept and the possible transport of raw materials and products. |
| Social parameters | Impacts on human health | Describes to what extent potential impairments of effects on human health can occur through the use of the concept. |
| Social parameters | Distribution effects: Benefits for a multitude of actors | Describes which actors can participate and benefit economically or otherwise, and who experiences economic strains. |
| Social parameters | Regional inclusiveness and participation | Describes whether the concept builds on existing structures, how qualified the jobs are that are created, and whether external expertise is needed or the expertise available locally. |
| Social parameters | Conflicts of use and resources | Describes whether the use of the concept is linked to potential conflicts of use and resources (impact on food security, large area requirement, critical resources, etc.), and what the scale of the conflicts is. |
| Social parameters | Ethical aspects | Determines whether ethical aspects (interference with nature, change in the landscape, religious values, distribution issues, etc.) are discussed as problematic. |

Annex 3: CO_2_ removal data

In this annex, the CO_2_ removal potentials are explained for each bio-based CDR concept. A summary overview of the two parameters for each concept for a starting point of implementing the concepts and for 2045 is given in Table A.2. While net greenhouse gas emissions data was also collected in the course of the project, it is not shown here due to large uncertainties and different system boundaries.

**Agriculture and soils.** Mineral soils of arable land in Germany have an average carbon content of 61 t ha^-1^ at a depth of 10 to 30 cm, while grassland soils have a content of 88 t ha^-1^ [33]. The highest CO_2_ removal potentials were found in agroforestry systems (5 - 20.8 t CO_2_ ha^-1^ a^-1^, depending on the choice of agroforestry system) [34]. The lowest CO_2_ removal potentials were found for applying organic fertiliser and compost (0.8 t CO_2_ ha^-1^ a^-1^) [35] and biochar (0.9 t CO_2_ ha^-1^ a^-1^) [36]. A combination of fertiliser with other concepts can result in a higher CO_2_ removal potential. All-year ground cover showed a CO_2_ removal potential of 1.2 t CO_2_ ha^-1^ a^-1^ [37], followed by no-till (2.1 t CO_2_ ha^-1^ a^-1^) [38] and land conversion from cropland to grassland (3.1 t CO_2_ ha^-1^ a^-1^) [1, 39].

These are usually average values that can be expected when applying the respective concept. How strong the potentials are, however, depends on which crop is cultivated, which soil and general climatic conditions prevail or, in the case of biochar, which pre-treatment is available and at which temperature it was produced [36].

**Peatland rewetting and paludiculture.** C accumulation after rewetting can be very high in the first 10-15 years after rewetting. After rewetting, vegetation builds up a layer that is biomass at the top and peat at the bottom, the acrotelm, which contains approx. 40 t C ha^-1^ after 20 years ([40] and additional unpublished measurements). This is a one-time C accumulation effect. After approx. 15 years, much lower C accumulation rates are assumed [41]. From year 16 on, the much lower long-term accumulation rate of 0.8 t CO_2_ ha^-1^ a^-1^ for natural wet peatlands is applied [42–44]. Since long-term measurements are missing, we assume that with elaborate water management, higher long-term rates are feasible. The temporal dynamic of the carbon accumulation after rewetting leads to CO_2_ removal values that require a timespan as a reference. Averaged for 20 years, the net CO_2_ removal potential results in 7.5 t CO_2_ ha^-1^ a^-1^ (averaged for 15 years: 9.7 t CO_2_ ha^-1^ a^-1^; averaged for 25 years: 6.1 t CO_2_ ha^-1^ a^-1^; averaged for 100 years: 2.14 t CO_2_ ha^-1^ a^-1^).

There is no or little data available for carbon accumulation rates in paludiculture because demonstration sites are too young. The yearly harvest and use of the biomass could lead to further substitution and net C removal (BECCS, long-lived building materials, biochar). We assume slightly lower CO_2_ removal rates than for peatland rewetting due to biomass harvest. Averaged for 20 years, those are approx. 4 t CO_2_ ha^-1^ a^-1^. New pilot sites with elaborate hydrological management show much higher removal rates (e.g., [45]).

The net C removal potential depends on the water level and site conditions. Current research results show that with optimised hydrological management, higher C removal rates are possible. This means that we need to link the CDR potential to the water level, which is sometimes difficult to implement in reality and only feasible at much higher costs. Due to site heterogeneity, only parts of a rewetted area are likely to have a net CDR effect. The real share depends on site conditions and investments in water management.

**Forest management practices.** Among the forestry measures investigated, afforestation is the one with the highest CO_2_ removal potential. Depending on the tree species, the annual CO_2_ removal through above-ground and below-ground tree biomass at an age of 20 years amounts to 1.1 - 9.6 t CO_2_ ha^-1^ a^-1^ (assuming moderate thinning, yield class 1 and a 10 % premium for the currently observable increase in growth rates, [11]). It is important to bear in mind that the various generations of yield tables differ considerably from one another in terms of their assumptions and results, and that newer yield tables (e.g., [46]) tend to indicate higher increments than in the past, due to improved growing conditions caused by atmospheric depositions and extended vegetation periods. Succession is an alternative way to establish new forests. Based on the example of a spruce stand established by succession on former grassland in Thuringia [47], it could be determined that the annual CO_2_ reduction through above-ground and below-ground tree biomass at an age of 20 years amounts to approx. 3-5 t CO_2_ ha^-1^ a^-1^ with moderate thinning, assuming yield classes 1 and 2 [11]. In the further course of time, growth rates continue to increase. However, the development of forest stands can also be impaired by calamities, which may reduce the accumulation of carbon stocks. By setting aside a forest stand, in principle, the carbon stock is conserved and no emissions are produced by harvesting or using wood as fuel [48]. Disadvantages, however, are the suboptimal increment, the loss of storage possibilities in harvested wood products, the loss of potential substitutions effects, and again the prolonged calamity risk. In set-aside beech stands in Hessen of about 120-170 years, the average annual above- and below-ground tree biomass growth (t ha^-1^ a^-1^) after 12-24 years turned out to be 19 % lower than in managed beech forests including harvesting [49]. If this 19 % is subtracted from the values given for the beech age classes 120, 140 and 160 years from [11], the remaining annual CO_2_ removal potential (current increment) can be determined to be approx. 14-16 t CO_2_ assuming today's set-aside beech stands of the age classes 120, 140 and 160 years in the public forest if no calamities occur [13]. The survival probability of 25-year-old beech stands at age 100 is 79 % according to recent studies that consider previous calamities. It is to be expected that the calamity-related threat to old stands will continue to increase in the future due to climate change and that the survival probability of stands older than 200 years in particular will thus decrease significantly [50].

**Long-lived biomass-based building materials.** In the case of long-lived biomass materials, the carbon removal potential is calculated considering a scale of 10,000 m^2^. When considering the increased use of engineered wood for scaling up market shares of wood construction, the main factors are the avoidance of upstream GHG emissions in particular in wood logistics and in adhesive and impregnation resin production. These determine to which degree the carbon uptake in wooden buildings is compromised by high GHG inventories in the production system of engineered wood products. Engineered wood products are assumed to be applied in multi-storey buildings as housing units for multi-family housing, whereas energetic renovation is assumed to be applied rather in single and multi-family housing but with no special incentive on building multi-storey buildings. Therefore, the potential for buildings with around 6 storeys is assumed to be around 2120 t CO_2_eq ha^-1^. When only considering one floor, this accounts for around 350 t CO_2_eq ha^-1^. The difference in CDR potential mainly results from height of multi-storey buildings and from suitability of materials for being used as structural load-bearing material.

Considering PyCCS or BCR-based CDR concepts, three fields of application are included into evaluating the specific concept. For the first field of application, which is the use of biochar-amended concrete elements, one major constraint is that these are rather used in non-structural, meaning non-loadbearing applications such as foundations, mega blocks and pavements, which limits the usage in multi-storey buildings in particular. Furthermore, for the second field of application, which is the use of biochar in green roof substrates, the composition of substrate mixtures and the depth of green roofs is a limiting factor for storing biochar as carbon substrate. Finally, the third field of application is the use of biochar in French drains. This is limited by the depth of these structures and by the potentials for unsealing the urban space and for developing water-sensitive settlement structures. Biochar-amended materials and insulation materials are normally not used in structural applications and are often dependent on retrofitting existing infrastructures whereas multi-storey buildings from wood construction might replace old inefficient buildings after their demolition with new highly efficient environmentally and climate-efficient building alternatives. The biochar-amended concrete materials are assumed to be excluded from structural applications due to limited height of buildings. Instead, they are preferably used in parking lots and pavement materials where no height is reached. Therefore, the potential ranges around 80–90 t CO_2_eq ha^-1^. The residual GHG emissions in 2045 from the upstream chains for all three concepts were assumed to be drastically reduced compared to 2020 according to assumptions from [51]. Just for the PyCCS-based concepts, higher residual emissions are to be expected in 2045 because no small BECCS installations for capturing CO_2_ emissions from pyrolysis were assumed. However, as these emissions are considered to be biogenic CO_2_, i.e., short cycle emissions, their global warming potentials can be evaluated in different ways considering contribution to global warming.

**Bioenergy with carbon capture and storage**. In the BECCS concepts, typical plant sizes are considered and their specific CO_2_ removal potential are related to one year. This means that larger bioenergy plants have a greater CO_2_ removal potential considering the expenses in the plant and the biomass used. When comparing the figures, these differences in plant size must be considered. CO_2_ removal potentials were estimated using average CO_2_ contents in the gas streams from the model bioenergy processes as well as literature values for capture rates. Against this background, among the concepts considered, the largest CO_2_ emissions can be removed at the large wood combustion plants.

**Summarised results.** In Table A.2, we show the CO_2_ removal potentials of the considered concepts for the starting point of implementation and for the year 2045. If the CO_2_ removal potentials are not available for the starting time in the literature, the averaged CO_2_ removal potential in the first 20 years, as stated in the factsheets, is used, except for the peatland concepts, where the first 15 years are chosen. It should be noted that the functional units for the CO_2_ removal potentials differ between the concepts. The natural sink enhancement concepts refer to one hectare, the BECCS concepts refer to a bioenergy plant of a fixed size defined in the factsheets and the building materials concepts refer to structural measures on an area of one hectare. This means that the systemic comparability of the land use-based and the more technical concepts is limited. It must be pointed out that the numbers presented in Table A.2 are rather uncertain. This is due to the high temporal and spatial dynamics of CO_2_ removal in the concepts. Choosing two time periods with the start of the implementation in 2045 is only exemplary and can only represent the real processes in a very simplified way.

**Table A.2** CO_2_ removal potentials in 2020 and in 2045 for the concepts considered

| **Bio-based CDR concept** | **CO_2_ removal potential 2020** | | **CO_2_ removal potential 2045** | |
| --- | --- | --- | --- | --- |
| **Agriculture and soils** | | In t CO_2_ ha^-1^ a^-1^ | | In t CO_2_ ha^-1^ a^-1^ |
| All-year ground cover | | -1.20 | | -0.51 |
| Organic fertiliser & compost | | -0.80 | | -0.80 |
| No-till | | -2.10 | | -2.10 |
| Land conversion | | -3.10 | | -12.00 |
| Agroforestry | | -20.80 | | -0.75 |
| Biochar | | -0.87 | | -0.87 |
| **Rewetting and paludiculture** | | In t CO_2_ ha^-1^ a^-1^ | | In t CO_2_ ha^-1^ a^-1^ |
| Rewetting | | -9.7 | | -6.1 |
| Paludiculture | | -4.8 | | -3.2 |
| **Forest management** | | In t CO_2_ ha^-1^ a^-1^ | | In t CO_2_ ha^-1^ a^-1^ |
| Afforestation Beech | | <-3.8 | | -3.8 |
| Afforestation Douglas fir | | <-9.6 | | -13.5 |
| Afforestation Oak | | <-1.1 | | -2.2 |
| Afforestation Scots pine | | <-4.8 | | -7.3 |
| Succession | | <-4.0 | | -5.8 |
| Permanent set-aside | | -15 | | -15 |
| Temporary set-aside | | -15 | | -15 |
| **Long-lived building materials** | | In t CO_2_ ha^-1^ | | In t CO_2_ ha^-1^ |
| Engineered wood products (min) | | -2120 | | -2120 |
| Engineered wood products (max) | | -2120 | | -2120 |
| Insulation materials (min) | | -285 | | -285 |
| Insulation materials (max) | | -315 | | -315 |
| PyCCS - concrete element | | -131 | | -131 |
| PyCCS - roof greening substrate | | -134 | | -134 |
| PyCCS - infiltration substrates in percolation troughs | | -896 | | -896 |
| **BECCS** | | In t CO_2_ a^-1^ | | In t CO_2_ a^-1^ |
| Bioethanol production (min) | | -378_,_899 | | -378_,_899 |
| Bioethanol production (max) | | -419_,_873 | | -419_,_873 |
| Biogas upgrading to biomethane (min) | | -6_,_547 | | -6_,_547 |
| Biogas upgrading to biomethane (max) | | -6_,_547 | | -6_,_547 |
| Biogas with CHP production (min) | | -3_,_196 | | -3_,_196 |
| Biogas with CHP production (max) | | -3_,_196 | | -3_,_196 |
| Combustion of paludi biomass (min) | | -1_,_703 | | -1_,_703 |
| Combustion of paludi biomass (max) | | -2_,_555 | | -2_,_555 |
| Combustion of woody biomass | | -2_,_990_,_000 | | -2_,_990_,_000 |
| Gasification | | -60_,_000 | | -60_,_000 |

Annex 4: Upscaling potential of the concepts to 1 million tons CO_2_ removed

For discussing upscaling dynamics, all concepts are required to provide a cumulative 1 million tons of CO_2_ removed over 25 years. These specifications allow us to calculate the resources associated with the deployment. The chosen timespan makes it possible to account for different temporal removal dynamics, while simultaneously looking at a politically relevant timeframe. This allows for better comparability. For the forestry concepts, however, it must be noted that 25 years cover only a minor part of usual rotation periods. We choose the ambitious removal target to discuss issues around a large-scale deployment, as required if climate targets are to be met. The value of 1 million tons is chosen arbitrarily, but staying in a sensible order of magnitude. Thus, a first impression of upscaling dynamics is provided to give an insight into the possible range of area and raw material requirements. The exact values are only comparable in a very limited way. The scaling results are shown in Table A.3. Before narrowing in on the concept areas, the analysis of technology readiness, infrastructure requirements, efficiency gains, and scale of deployment required for large-scale implementation is presented.

**Technology readiness and infrastructure requirements.** Most technologies and feedstock provisioning of the concepts are well established, which is reflected in a high technology readiness level (TRL 7-9 for most concepts). In addition, most of the concepts can be integrated in existing infrastructure with only few adaptations. For individual agricultural businesses, an adaptation of machinery and mode of operation might be necessary. The infrastructure needed for rewetting drained peatlands depends on the peatland type and site conditions. For the forestry concepts, there is no need for intensive infrastructure. For building materials, the infrastructure need depends on the used resource, with PyCCS concepts having the highest infrastructure requirements. BECCS concepts have comparably high infrastructure needs, as CO_2_ transportation is not yet well-established, and feedstock and water supply have to be ensured.

**Efficiency gains.** Improvements in CO_2_ capture efficiency are currently under investigation for many of the bio-based CDR concepts. With many of the concepts being related to creating or maintaining natural sinks, the possibility to change processes for enhanced efficiency is limited. Rather, increases in efficiency result from value chain integration and combination with other concepts.

**Scale of deployment.** Current and future deployment depends on many factors such as site selection (e.g., for paludiculture or construction projects), investment decisions, and biomass mobilisation rates (e.g., for building materials and bioenergy plants). Bottlenecks differ for the concepts, i.e., constraints in technical capacities, land availability, or legal frameworks might be critical.

**Agriculture and soils.** The agricultural concepts have a high capacity for upscaling, as most agricultural land is degraded and the concepts could store large amounts of CO_2_ in the soil. For all concepts related to natural sink enhancement, a small fraction of the available area in Germany would be needed for deploying the CDR concept at a scale of removing 1 million t CO_2_. Depending on the concept, the areas for soil management practices range between 48,000 and 1.2 million ha, with a total agricultural area of 9,953,000 ha in Germany [52]. In order to get an idea of what would be needed to achieve certain climate goals, such upscaling is useful. However, it is difficult to predict how feasible this is in practice, as mineral soils can also quickly become saturated and the potential can quickly drop to zero. Furthermore, the durability of carbon storage in agricultural soils is still difficult to predict, as the soils - depending on the respective concept - are regularly tilled.

**Peatland rewetting and paludiculture.** Considering the almost 1.8 million ha drained organic soils, there is a very large potential area for emission reduction and additionally for CDR through scaling up peatland rewetting. We would need around 1,300 ha of CDR-oriented peatland rewetting to achieve 1 Mt CO_2_ removal within 25 years (660 ha for a 50-year perspective). However, land use competition limits the upscaling potential. We cannot deduce on which peatland areas CDR is possible and how much more peatland area needs to be rewetted in order to exploit the CDR potential. The actual scale of deployment is also dependent on investment decisions.

**Forest management practices.** The implementation possibilities for forest management depend on a plethora of assumptions (e.g. availability of land for afforestation, previous land use, tree species and tree growing conditions). Assuming good growing conditions and sufficient land availability, an afforestation with Douglas fir, for example, would sequester a total of 1 Mt of CO_2_ on approx. 3,000 ha over a time span of 25 years in Germany. Under weaker growing conditions, the time required would be correspondingly higher. Competition for land use and wood resources limits the upscaling potential: Afforestation and succession withdraw land from agricultural use and set-aside forests cannot be used for wood production.

**Long-lived biomass-based building materials.** When investigating the upscaling of building material CDR concepts, a proper site selection is crucial especially for wood constructions, considering, e.g., shortage in available housing units and regional constraints for providing housing for an affordable living. The highest potential for CDR is expected from multi-storey houses. However, planning constraints have to be taken into account. For insulation materials, the scaling to 1 Mt is beneficial as a contribution to more energy-efficient buildings through energetic renovation, which leads to lower heating requirements. On top of that, the use of agricultural residues can be a value-added for agricultural upstream chains. A maximum scale-up for energetic renovation could be possible in a rather linear manner of 40,000 ha per year for the next 25 years, as the maturity of insulation materials is high, the production capacities are given in many European countries and the demand can be covered from the different production plants. A minor fraction of the available areas (0.04 % for biochar applications, 3 % for renewable insulation materials) would be needed to achieve a CO_2_ removal of 1 Mt. The provision of wood and agricultural biomass as well as building regulations are limiting factors for the upscaling potential.

**Bioenergy with carbon capture and storage**. The upscaling for BECCS plants is difficult to compare on an area basis because of the system boundary assumptions on retrofitting, on using residues where possible which do not come with additional area requirements, and on underground storage. Therefore, the number of BECCS plants was selected as a comparison base. For the large-scale model plants on wood combustion, wood gasification and sugar beet fermentation for bioethanol production, only a fraction of one plant needs to operate for 25 years to achieve a 1 Mt CO_2_ removal. However, those plants are fuelled by energy crops, which would require an area for wood pellets for combustion, for wood chips for gasification, and for sugar beet for bioethanol production. The scale-up is therefore dependent on area availability and related trade-offs such as feedstock supply and competition with alternative uses (cf. Annex 5). The smaller-scale BECCS model plants (a biogas upgrading plant, a biogas CHP plant, and a heat plant operating on paludi biomass) require a number of 6, 13, and around 36 plants to remove 1 Mt CO_2_ over 25 years, respectively. One main challenge is the integration of these small and dispersed CO_2_ point sources into the CO_2_ transport infrastructure. However, recent publications increasingly emphasise the system benefits and opportunities of small-scale BECCS [53–55].

**Table A.3** Resources needed for upscaling the concepts to 1 million tons of CO_2_ removal in 25 years within the system boundaries. A) Values for concepts with area-based system boundaries. B) Values for concepts with a bioenergy plant-based system boundary

| 1. **Area-based CDR concept** | | **Cumulative CO_2_ removal potential in t CO_2_ ha^-1^** | | **Number of hectares to achieve 1 Mt CO_2_ removal** | | **Share of reference area for CO_2_ removal in %** | **Reference area [as of 2020]** | **Raw material demand** | |  |
| --- | --- | --- | --- | --- | --- | --- | --- | --- | --- | --- |
| **Agriculture and soils** | |  | |  | |  |  |  | |  |
| All-year ground cover | | 14.3 - 34.3 | | 69,930 - 29,138 | | 0.7 - 0.3 | of agricultural area in Germany (9,953,000 ha) | n/a | |  |
| Organic fertiliser & compost | | 20.8 | | 48,077 | | 0.5 | of agricultural area in Germany (9,953,000 ha) | n/a | |  |
| No-till | | 54.6 | | 18,315 | | 0.2 | of agricultural area in Germany (9,953,000 ha) | n/a | |  |
| Land conversion | | 277 | | 3,610 | | 0.04 | of agricultural area in Germany (9,953,000 ha) | n/a | |  |
| Agroforestry | | 65 | | 15,385 | | 0.2 | of agricultural area in Germany (9,953,000 ha) | n/a | |  |
| Biochar | | 22.6 | | 44,248 | | 0.44 | of agricultural area in Germany (9,953,000 ha) | n/a | |  |
| **Rewetting and paludiculture** | |  | |  | |  |  |  | |  |
| Rewetting | | 153.6 (750)^1^ | | 6,510 (1,333)^1^ | | 0.4 (0.07)^1^ | of area of organic soils in Germany (1,822,109 ha) | n/a | |  |
| **Forest management** | |  | |  | |  |  |  | |  |
| Afforestation Beech | | < 113 | | 10,526 | | 0.03 | of total German area (35,758,800 ha) | 52,630,000 trees | |  |
| Afforestation Douglas fir | | 337 | | 2,963 | | 0.01 | of total German area (35,758,800 ha) | 5,926,000 trees | |  |
| Afforestation Oak | | 55.8 | | 18,182 | | 0.05 | of total German area (35,758,800 ha) | 127,274,000 trees | |  |
| Afforestation Scots pine | | 219.9 | | 5,479 | | 0.02 | of total German area (35,758,800 ha) | 16,437,000 trees | |  |
| Succession | | 110 - 190 | | 6,957 | | 0.02 | of total German area (35,758,800 ha) | 1,391,400 trees | |  |
| Permanent set-aside | | n/a | | 2,640 | | 1.29 | of potential set-aside area in Germany (205,200 ha) | n/a | |  |
| Temporary set-aside | | n/a | | 2,640 | | 1.29 | of potential set-aside area in Germany (205,200 ha) | n/a | |  |
| **Long-lived building materials** | |  | |  | |  |  |  | |  |
| Engineered wood products | | 53000 | | 472 | | 0.08 | of new constructions of multi-family houses in Germany (1306 ha a^-1^) | n/a | |  |
| Insulation materials | | 2700 | | 9,259 | | 3 | of average area energetically renovated in Germany (13,011 ha a^-1^) | n/a | |  |
| PyCCS | | 18000 | | 1,389 | | 0.04 | of German traffic and settlement area (3,190,000 ha) | n/a | |  |
| 1. **BECCS** | **Cumulative CO_2_ removal potential in t CO_2_** | | **Number of plants to achieve 1 Mt CO_2_ removal** | | **Biomass demand for 1 Mt CO_2_ removal in t fresh matter (tFM)** | | **Other raw material demand for 1 Mt CO_2_ removal in t** | **Energy demand for 1 Mt CO_2_ removal in GJ** | | |
| Bioethanol production | 9,472,476 - 10,496,832 | | 0.1 | | 5,410,412 (sugar beet) | | 779 - 3722 (yeast)  7358 - 9955 (coke)  125,522 - 268,356 (limestone)  199 - 520 (activated carbon) | 533,715 (electrical)  1,027,978 (steam)  2,785,064 (fuel) | | |
| Biogas upgrading to biomethane | 163,675 | | 6 | | 7,131,816 (maize silage) | | 351 - 993 (activated carbon) | 311,746 (electrical)  0 (steam)  2,785,092 (fuel) | | |
| Biogas with CHP production | 79,900 | | 13 | | 2,304,130 (cattle slurry)  921,464 (solid cattle manure)  1,382,353 (wheat straw) | | 800 (MEA, 30 %)  75 (activated carbon) | 362,713 (electrical)  3,199,950 (steam)  2,784,559 (fuel) | | |
| Combustion of paludi biomass | 23,376 - 35,065 | | 29 - 43 | | 855,578 (round bales) | | 800 (MEA, 30 %)  75 (activated carbon) | 362,713 (electrical)  3,199,950 (steam)  2,784,559 (fuel) | | |
| Combustion of woody biomass | 74,750,000 | | 0.01 | | 856,187 (wood pellets) | | 800 (MEA, 30 %)  75 (activated carbon) | 362,713 (electrical)  3,199,950 (steam)  2,784,559 (fuel) | | |
| Gasification | 1,500,000 | | 0.7 | | 2,730,367 (dry wood chips) | | 800 (MEA, 30 %)  75 (activated carbon) | 362,713 (electrical)  3,199,950 (steam)  2,784,559 (fuel) |  |  |

Annex 5: Outlook on the deployment of the concepts

From a carbon pool perspective, the starting points and the end-points of the presented CDR methods differ significantly considering their current levels and future ramp-up. This regards both their calibration against data series from the past [56, 57] as well as when envisioning a future state of a higher equilibrium [58]. In this state, carbon pools would tend toward their saturation and technology-driven CDR methods would reach a continuous removal capacity deployed in full scale. For natural sinks, carbon pools that were diminished by poor management practices in the past will be replenished with regenerative practices which CDR concepts can provide, such as soil organic carbon sequestration or CDR potentials in peatland restoration. Other carbon pools were non-existent or underdeveloped in the past such as full-scale carbon storage in the building sector. With modern architecture taking over and urbanisation accelerating, there is a potential to increase the overall amount of carbon stored in the German and international building stock. Other carbon pools are well maintained such as the carbon storage in forest ecosystems (e.g., [59]), but future best practices can support the exploitation of further significant CDR potentials in forest management. Further potentials also lie in facilitating the retrofitting of bioenergy plants towards deploying BECCS process technologies in the near future [60].

**Agriculture and soils.** In terms of soil C sequestration, part of its attractiveness as a bio-based CDR strategy is that carbon stocks are mostly depleted on the land currently used for agriculture, so this approach to sequestering CO_2_ does not require a land use conversion (e.g. to forests) or competition for land resources. Furthermore, increasing soil organic matter or C content is highly beneficial for soil health and fertility [1].

The CDR concepts presented for agriculture have a humus-building effect primarily through the increased input of organic carbon. Their potential for carbon enrichment in soils is limited both in terms of quantity and time, as a new equilibrium between input and mineralisation of organic carbon is established. Accordingly, the climate protection effect through the additional binding of CO_2_ carbon in the soil is also limited in time. If the humus-building measure is terminated, the previously enriched carbon stocks are quickly lost again. The correlations make it clear that climate protection through humus formation is limited in time and also requires continuity [1, 33, 61, 62].

There is a variety of C sequestration practices that can be applied, and the best solutions vary depending on climate, soil and farming practices. Many practices (e.g. improvements in crop rotation, cover crops use, changes in tillage, fertiliser management) have already been developed and their effectiveness is relatively well studied. The widespread introduction of such measures could be quite rapid. Other potential practices, such as large-scale use of soil amendments like biochar, require additional research and development to improve economic feasibility and to address fears and prejudices against biochar application [1]. It also seems to be unclear how long carbon is ultimately stored in agricultural soils. Comprehensive long-term experiments and soil monitoring could provide clarification on that.

**Peatland rewetting and paludiculture.** Peatland rewetting combined with paludiculture could offer a site-adapted land use alternative. The biomass can be used for a wide range of traditional and innovative food, feed, fibre and fuel products [63]. Peatland rewetting without productive use will lead to a loss of agricultural value creation in rural areas, connected to the loss of jobs or a change in value creation. The net C removal and net GHG emissions are derived from rewetting projects that were implemented for nature conservation. The maximal potential for CDR through peatland rewetting could probably increase by improved water management. More research and best-practice examples are necessary to have a better understanding of the C accumulation dynamics after rewetting and the influence of water management, choice of crop and other site conditions.

Peatland rewetting and paludiculture are at first an important and necessary measures for the reduction of the high GHG emissions from drained organic soils (53 million t CO_2_eq a^-1^, [64]). They have the additional potential to permanently sequester C in biomass and peat. Peatland rewetting contributes to several ecosystem services: improvements for the landscape hydrology (water retention, groundwater recharge, [65]), flood protection and buffer during heavy rainfall, nutrient retention, local cooling effects, and biodiversity [66, 67]. The impacts depend on the initial conditions. In most cases, large improvements can be expected. In addition, tourism could benefit.

For the paludiculture concept, it was not possible to include the biomass use and related calculations within the project time. Proper life cycle assessments for paludiculture products need to be done in future research activities to analyse the whole CDR and GHG avoidance potential of paludiculture.

Rewetting poses inevitably a conflict with current land use. A change in the current land use and change of the landscape is unavoidable. Paludiculture is not yet fully researched and demonstrated. There are still risks for the pioneers, e.g., in long-term crop management and profitability. Existing technical challenges and other uncertainties as well as the agricultural policy framework currently make it unattractive to invest in new branches of operation. Through education, transparency and participation of local residents, acceptance should be gained and solutions for potential conflicts should be negotiated. The framework conditions must be adapted and income alternatives (e.g. through payment for ecosystem services, other incentives, new value chains for paludiculture products) must be established [68]. There are many conflicts and barriers for the land use change towards paludiculture, because there are currently risks and missing incentives for farmers [68]. Rewetting without use has currently even lower acceptance because of a missing perspective for land users.

**Forest management practices.** Afforestation with different tree species as well as succession enable the development of adaptable mixed forests, which makes long-term carbon sequestration possible [69]. According to the Federal Forestry Act, afforestation leads to a permanent change in land use which prohibits returning to other land uses in the future [70]. In this sense, the carbon stock built up within the newly established forests is even permanent. Furthermore, harvested wood products store carbon even after the timber has eventually been harvested, and the substitution potential of wood utilisation can also lead to emission savings in other sectors [71]. Depending on the type of area to be afforested, the local site factors must be considered so that the most suitable and healthy tree species combination can be selected. An advantage of new forest areas is the positive social perception and the willingness to pay for additional afforestations from the population, which can facilitate the implementation of the measure [72, 73]. In contrast to afforestation, allowing natural succession is a more cost-effective alternative. Both stand establishment costs and the development of a specific infrastructure or labour costs for maintenance measures are avoided [74]. In addition to its low resource demands, it can take place both on rural land, agricultural land or urban fallow land [75].

A disadvantage of afforestation and succession is the increased vulnerability to damages. Extreme climatic changes can lead to stagnation of succession, especially in the early stages. Devastation due to droughts, storms, forest fires or browsing by game pose a considerable risk to stand establishment [50]. Another disadvantage is that afforestation and succession lead to competition for land use with potential housing in urban areas or on agricultural land. The result is increased opportunity costs due to land value losses. Forest owners who hold the property rights to a developing forest area could be restricted in their future economic use by forest law as well as by nature conservation laws [76].

Permanent and temporary set-aside are short-term but very efficient measures to maintain storage in the standing stocks and, to a small extent, CO_2_ accumulation. A consideration period of approx. 25-30 years is assumed here [13]. In this context, permanent set-asides in particular do not cause any expenditure through labour or infrastructure because management is excluded, but high opportunity costs, because the use of the accumulated timber stock capital is waived.

**Long-lived biomass-based building materials.** The CDR concepts for increasing carbon storage in buildings and in the built environment offer many co-benefits besides the CDR potentials, such as environmentally and climate-friendly housing operation, strong potentials for GHG abatement, further benefits for biodiversity such as green roof habitats and benefits for extreme weather adaptation such as mitigation of drought impacts on urban trees and strengthening of heavy rain infiltration into urban soils. Furthermore, the trends of rapid urbanisation and of energetic renovation offer precious timing benefits for integrating CDR strategies into current global megatrends for ramping up CDR in the built environment fast-paced and near to exponentially in the first half of the century.

However, old path dependencies and new resource lock-ins are abundant as the building sector is a very slow-paced, long-term committed sector with high system inertia. The energetic renovation requires a high commitment of housing owners for fast-paced investments into energetic renovation to increase building efficiency, therefore the CDR potential is rather a co-benefit. Regarding the application of biochar in urban environments, the initiatives for transforming sealed cities with compacted soils and high run-off capacities into sponge cities with high infiltration capacities require long-term planning, strong incentives and the harnessing of multiple co-benefits for ensuring attractive amortisation times in urban infrastructure investments.

An equilibrium with a constantly high level of carbon stored in the built environment might be reached by the end of the century as global population and settlement structures might be much more static and less expanding [77], along with an end of the phase of rapid urbanisation. This would reduce the potential for additional CDR through building materials.

To support a fast ramp-up, the accounting for embodied carbon and for embodied energy should be included into financing incentives for new building construction as well as for loans for energetic renovation. In particular the use of biomass-based insulation materials is part of a persisting market niche, but which could benefit from strong incentives so that carbon storage is coupled with a more energy-efficient building operation. When planning sponge city concepts, the strengthening of infiltration capacities should be planned in line with enhanced carbon sequestration, e.g., by including PyCCS innovations into extreme weather adaptation measures.

Envisioning integrated supply chains for feedstock provision for biochar production such as using prunings from urban trees and landscape clearance biomass from other urban blue-green infrastructures for biochar pyrolysis can further support implementing self-reinforcing and cross-fertilizing systems for sponge cities.

In terms of construction materials, not all are suitable for reuse in different building envelopes (e.g., loose fill-in materials). The increase of modular building elements needs to be strengthened also in energetic renovation and reuse quota should be mandatory so that incineration is not the standard end-of-life path for insulation materials but that instead, strong incentives for refurbishment and reuse are provided.

**Bioenergy with carbon capture and storage**. In many studies BECCS is described as an option with high potential, because it combines renewable energy provision with CDR [78]. In Germany there is the unique situation with already more than 9600 biogas plants in place. Especially the currently running 240 biomethane provision plants (as well as 13 bioethanol plants) already have a CO_2_ separation in place, as it is necessary for providing high-quality biomethane and bioethanol [79]. Pooling of biogas plants and upgrading the produced biogas to biomethane is also under discussion, because biomethane is requested in the transport sector under the advanced biofuels support scheme, which was implemented under the Renewable Energy Directive II [79], as well as higher demands in other energy sectors and as a resource base for the chemical sector [80, 81]. Moreover, former coal plants after the phase-out are under discussion to be retrofitted to run on woody biomass combined with CCS [82, 83]. Additionally, there are novel options for CDR via biogas and biochar currently under development [84, 85].

The main bottleneck for BECCS is biomass availability. Biogas plants are currently mainly fed with energy crops such as maize silage, grass silage etc. [86]. Under the consideration of increasing demands of land for food security, biodiversity restoration and also nature-based solutions for GHG reduction, it is expected that the land availability for purpose-grown crops will decrease and biogas feedstocks will increasingly be residues, wastes and biomass from the adopted land use (blowing strips, paludicultures etc.). Currently it is not clear if this transformation of the resource base is successful and how many biogas or biomethane plants will be in place in the mid-term [87]. The issue of resource availability also impacts the ligoncellulosic-based power plants. Because of the necessary amounts and the location of the coal power plants, increasing wood imports can be expected when shifting the resource base of those plants. Risks of sustainable sourcing and also price uncertainties may hinder this transformation. Additionally, the value of those plants in a more and more renewable power system is limited [88]. Finally, all BECCS concepts currently lack a national carbon capture and storage strategy and the permission to store CO_2_ geologically [89].

To bring BECCS into the market in Germany, it is necessary to specify the national carbon capture and storage strategy, including targets, incentives but also concepts of CO_2_ capture and transport infrastructure. Additionally, a clarification on national storage of CO_2_ in geological formations is necessary. Once the legal framework is established, envisioning integrated supply chains for potential BECCS feedstocks is key. This includes a better integration of bioenergy plants in cascades and agricultural circular economy approaches [90], but also using urban residues such as prunings from urban trees and landscape clearance biomass from other urban blue-green infrastructures. Also, combined concepts such as integrating biogas with biochar production can enhance the CO_2_ removal per unit biomass significantly [91].

Annex 6: Longlist of bio-based CDR concepts and application of concept selection criteria

Table A.4 presents the collection of possible bio-based CDR concepts in a longlist and their selection. Additionally, the creation of distinct concepts is made transparent by indicating where separate concepts from the longlist were merged to one concept.

**Table A.4** Longlist of possible bio-based CDR concepts and concept selection

| **Bio-based CDR concept** | **Application of concept selection criteria** | |
| --- | --- | --- |
| **Agriculture and soils** | |  |
| All-year ground cover | | meets all criteria |
| Organic fertiliser & compost | | meets all criteria |
| No-till | | meets all criteria |
| Land conversion | | meets all criteria |
| Agroforestry | | meets all criteria |
| Biochar | | meets all criteria |
| **Rewetting and paludiculture** | |  |
| Rewetting | | meets all criteria |
| Paludiculture | | meets all criteria |
| Paludiculture through harvesting naturally occurring biomass (wet meadows) from already wet or rewetted peatlands | | Considered under "paludiculture" |
| Paludiculture through rewetting, establishment and harvesting of biomass from cultivation crops (reeds, cattails, peat moss) | | Considered under "paludiculture" |
| Paludiculture through rewetting, establishment and harvesting of biomass from | | Considered under "paludiculture" |
| Paludiculture through rewetting, establishment and harvesting of biomass from forestry crops (black alder) | | Considered under "paludiculture" |
| Use of peat moss biomass as a peat substitute | | No long-term CO_2_ removal |
| Production of fibres for paper, cardboard, and moulds | | No long-lived product, thus no long-term CO_2_ removal |
| Lignocellulosic biorefinery for production of platform chemicals for reusable packaging | | No long-lived product, thus no long-term CO_2_ removal |
| Building and insulating materials ( reed, cattail, wood) | | Considered under "long-lived building materials" |
| **Forest management** | |  |
| Afforestation Beech | | meets all criteria |
| Afforestation Douglas fir | | meets all criteria |
| Afforestation Oak | | meets all criteria |
| Afforestation Scots pine | | meets all criteria |
| Succession | | meets all criteria |
| Permanent set-aside | | meets all criteria |
| Temporary set-aside | | meets all criteria |
| Substitution potentials through wood product storage extension in material and energetic use | | Material use considered under "Engineered wood products", Energetic use is a CO_2_ reduction measure not removal |
| Underground wood storage | | Not a forest management measure, but a subsequent wood use measure which is only indirectly influenced by forest management practices |
| Thinning regime | | Low effectiveness compared to selected concepts; high hurdles for practical implementation (e.g., workforce availability) |
| Rotation period control | | Considered under permanent and temporary set-aside |
| Technical damage control | | Not a regular forest management measure, but a wood use measure subsequent to the occurrence of a risk event |
| Natural damage control (Deadwood as CO_2_ storage, litter layer and soil) | | Not a regular forest management measure, but a wood use measure subsequent to the occurrence of a risk event |
| **Long-lived building materials** | |  |
| Engineered wood products | | meets all criteria |
| Insulation materials | | meets all criteria |
| PyCCS - concrete element | | meets all criteria |
| PyCCS - roof greening substrate | | meets all criteria |
| PyCCS - infiltration substrates in percolation troughs | | meets all criteria |
| Natural fibre-based composite materials - hempcrete | | Currently no relevant potential for Germany due to lacking market relevance (no established value chains for feedstock provision, processing and construction) |
| Natural fibre-based composite materials - mycel-based materials | | TRL insufficient for insect and mould infection protection within service lifetime; low potential compared to insulation materials (which compete for the same feedstock) |
| Straw products, e.g., in straw bale construction or for straw clay plastering | | Currently no relevant potential for Germany due to high market reluctance (high fire safety risk connected to risk of reversal of CO_2_ storage) |
| Bamboo-based systems, e.g., biochar from bamboo | | Relying on imported biomass and therefore excluded from analysis for Germany |
| Fibres or bricks from macroalgae | | TRL under 6 for alagae farming and harvesting as well as processing and final product solutions; currently relying on imported biomass |
| Bioconcrete and self-healing bioconcrete | | TRL under 6 (biotechnological process and further processing steps) |
| Biocement | | TRL under 6 (biotechnological process and further processing steps) |
| **BECCS** | |  |
| Bioethanol production | | meets all criteria |
| Biogas upgrading to biomethane | | meets all criteria |
| Biogas with CHP production | | meets all criteria |
| Combustion of paludi biomass | | meets all criteria |
| Combustion of woody biomass | | meets all criteria |
| Gasification | | meets all criteria |
| Biowaste combustion with CHP production | | Considered under "combustion of woody biomass" |
| Gasification with subsequent synthesis of synthetic natural gas | | Considered under "gasification" |
| Algae-based BECCS | | Considered under the anaerobic digestion concepts "biogaswith CHP production" and "Biogas upgrading to biomethane" |
| Pyrolysis | | Considered under "PyCCS" |

References

1. Paustian, K., Larson, E., Kent, J., Marx, E. & Swan, A. Soil C sequestration as a biological negative emission strategy. *Front. Clim.* **1**, 1244; 10.3389/fclim.2019.00008 (2019).

2. Luo, Z., Wang, E. & Sun, O.J. Can no-tillage stimulate carbon sequestration in agricultural soils? A meta-analysis of paired experiments. *Agr. Ecosyst. Environ.* **139**, 224; 10.1016/j.agee.2010.08.006 (2010).

3. Paustian, K. Carbon sequestration in soil and vegetation and greenhouse gases emissions reduction. *Glob. Environ. Change*, 399; 10.1007/978-94-007-5784-4_10 (2014).

4. Six, J., Conant, R.T., Paul, E.A. & Paustian, K. Stabilization mechanisms of soil organic matter: implications for C-saturation of soil. *Plant Soil* **241**, 155; 10.1023/A:1016125726789 (2002).

5. Barthelmes, A.*, et al.* Evaluierung von Moor-Wiedervernässungen in Deutschland – Ergebnisse, Erfahrungen und Empfehlungen. *Naturschutz Biol. Vielfalt*, 121 (2021).

6. Joosten, H., Tanneberger, F. & Moen, A. Mires and peatlands of Europe: Status, distribution and conservation, 730 (2017).

7. Joosten, H., Sirin, A., Couwenberg, J., Laine, J. & Smith, P. The role of peatlands in climate regulation in *Peatland Restoration and Ecosystem Services*, (ed. Bonn, A., Allott, T., Evans, M., Joosten, H. & Stoneman, R.) 63 (Cambridge University Press, 2016).

8. Ziegler, R.*, et al.* Wet peatland utilisation for climate protection – An international survey of paludiculture innovation. *Clean. Eng. Technol.* **5**, 100305; 10.1016/j.clet.2021.100305 (2021).

9. Moog, M. & Bösch, M. Interest rates in the German forest valuation literature of the early nineteenth century. *For. Policy Econ.* **30**, 1; 10.1016/j.forpol.2013.03.004 (2013).

10. Wüstemann, H.*, et al.* Synergies and trade-offs between nature conservation and climate policy: Insights from the “Natural Capital Germany – TEEB DE” study. *Ecosyst. Serv.* **24**, 187; 10.1016/j.ecoser.2017.02.008 (2017).

11. Paul, C., Weber, M. & Mosandl, R. Kohlenstoffbindung junger Aufforstungsflächen. *Karl Gayer Institut*, 47 (2009).

12. Bösch, M.*, et al.* Forest ecosystem services in rural areas of Germany: Insights from the national TEEB study. *Ecosyst. Serv.* **31**, 77; 10.1016/j.ecoser.2018.03.014 (2018).

13. Bolte, A.*, et al.* Einschlagstopp in alten, naturnahen Buchenwäldern im öffentlichen Besitz, Report. https://ageconsearch.umn.edu/record/322043/files/dn065056.pdf (2022).

14. Amiri, A., Ottelin, J., Sorvari, J. & Junnila, S. Cities as carbon sinks—classification of wooden buildings. *Environ. Res. Lett.* **15**, 94076; 10.1088/1748-9326/aba134 (2020).

15. Churkina, G.*, et al.* Buildings as a global carbon sink. *Nat. Sustain. (Nature Sustainability)* **3**, 269; 10.1038/s41893-019-0462-4 (2020).

16. Pittau, F., Krause, F., Lumia, G. & Habert, G. Fast-growing bio-based materials as an opportunity for storing carbon in exterior walls. *Build. Environ.* **129**, 117; 10.1016/j.buildenv.2017.12.006 (2018).

17. Pomponi, F., Hart, J., Arehart, J.H. & D’Amico, B. Buildings as a global carbon sink? A reality check on feasibility limits. *One Earth* **3**, 157; 10.1016/j.oneear.2020.07.018 (2020).

18. Minx, J.C.*, et al.* Negative emissions—Part 1: Research landscape and synthesis. *Environ. Res. Lett.* **13**, 63001; 10.1088/1748-9326/aabf9b (2018).

19. Andersson, J., Tamm, D. & Berg, K. BECCS from biogas production. *RISE Rapport*, **92**. https://www.diva-portal.org/smash/get/diva2:1602919/FULLTEXT01.pdf (2021).

20. Bui, M., Di Zhang, Fajardy, M. & Mac Dowell, N. Delivering carbon negative electricity, heat and hydrogen with BECCS – Comparing the options. *Int. J. Hydrogen Energy* **46**, 15298; 10.1016/j.ijhydene.2021.02.042 (2021).

21. Emenike, O.*, et al.* Initial techno-economic screening of BECCS technologies in power generation for a range of biomass feedstock. *Sustain. Energy Technol. Assess.* **40**, 100743; 10.1016/j.seta.2020.100743 (2020).

22. Friman, M. (ed.). Bioenergy with carbon capture and storage (European Liberal Forum, 2018).

23. Fuss, S. & Johnsson, F. The BECCS implementation gap–a Swedish case study. *Front. Energy Res.* **8**, 298; 10.3389/fenrg.2020.553400 (2021).

24. Negri, V.*, et al.* Life cycle optimization of BECCS supply chains in the European Union. *Appl. Energy* **298**, 117252; 10.1016/j.apenergy.2021.117252 (2021).

25. Otto, D., Pfeiffer, M., Brito, M.M. de & Gross, M. Fixed Amidst Change. *Sustainability* **14**, 7342; 10.3390/su14127342 (2022).

26. Hilaire, J.*, et al.* Negative emissions and international climate goals—learning from and about mitigation scenarios. *Clim. Change* **157**, 189; 10.1007/s10584-019-02516-4 (2019).

27. Prado, A., Chiquier, S., Fajardy, M. & Mac Dowell, N. Assessing the impact of carbon dioxide removal on the power system. *iScience* **26**, 106303; 10.1016/j.isci.2023.106303 (2023).

28. ZEP. Biomass with CO2 capture and storage (Bio-CCS). The way forward for Europe. https://network.bellona.org/content/uploads/sites/3/EBTP__ZEP_Report_Bio-CCS_The_Way_Forward.pdf (2012).

29. NEGEM. Stocktaking of scenarios with negative emission technologies and practices - Documentation of the vision making process and initial NEGEM vision. https://www.negemproject.eu/wp-content/uploads/2021/02/NEGEM_D_8_1-1.pdf (2021).

30. Forster, J., Vaughan, N.E., Gough, C., Lorenzoni, I. & Chilvers, J. Mapping feasibilities of greenhouse gas removal. *Glob. Environ. Change* **63**, 102073; 10.1016/j.gloenvcha.2020.102073 (2020).

31. Stavrakas, V., Spyridaki, N.-A. & Flamos, A. Striving towards the deployment of bio-energy with carbon capture and storage (BECCS). *Sustainability* **10**, 2206; 10.3390/su10072206 (2018).

32. Global CCS Institute. Bioenergy and carbon capture and storage. https://www.globalccsinstitute.com/wp-content/uploads/2019/03/BECCS-Perspective_FINAL_18-March.pdf (2019).

33. Flessa, H.*, et al.* Humus in landwirtschaftlich genutzten Böden Deutschlands. Ausgewählte Ergebnisse der Bodenzustandserhebung. https://www.bmel.de/SharedDocs/Downloads/DE/Broschueren/Bodenzustandserhebung.pdf?__blob=publicationFile&v=10 (2019).

34. Tsonkova, P. & Böhm, C. CO2-Bindung durch Agroforst-Gehölze als Beitrag zum Klimaschutz. *Loseblatt*, **6**. Forschungsprojekt "Innovationsgruppe AUFWERTEN – Agroforstliche Umweltleistungen für Wertschöpfung und Energie". https://agroforst-info.de/wp-content/uploads/2021/02/06__CO2-Bindung.pdf (2020).

35. Körschens, M.*, et al.* Effect of mineral and organic fertilization on crop yield, nitrogen uptake, carbon and nitrogen balances, as well as soil organic carbon content and dynamics: results from 20 European long-term field experiments of the twenty-first century. *Arch. Agron. Soil Sci.* **59**, 1017; 10.1080/03650340.2012.704548 (2013).

36. Schmidt, H.-P., Hagemann, N., Abächerli, F., Leifeld, J. & Bucheli, T. Pflanzenkohle in der Landwirtschaft. Hintergründe zur Düngerzulassung und Potentialabklärung für die Schaffung von Kohlenstoff-Senken (2021).

37. Poeplau, C. & Don, A. Carbon sequestration in agricultural soils via cultivation of cover crops – A meta-analysis. *Agric. Ecosyst. Environ.* **200**, 33; 10.1016/j.agee.2014.10.024 (2015).

38. West, T.O. & Post, W.M. Soil organic carbon sequestration rates by tillage and crop rotation. *Soil Sci. Soc. Am. J.* **66**, 1930; 10.2136/sssaj2002.1930 (2002).

39. Conant, R.T., Cerri, C.E.P., Osborne, B.B. & Paustian, K. Grassland management impacts on soil carbon stocks: a new synthesis. *Ecological applications : a publication of the Ecological Society of America* **27**, 662; 10.1002/eap.1473 (2017).

40. Mrotzek, A., Michaelis, D., Günther, A., Wrage-Mönnig, N. & Couwenberg, J. Mass Balances of a Drained and a Rewetted Peatland: on Former Losses and Recent Gains. *Soil Syst.* **4**, 16; 10.3390/soilsystems4010016 (2020).

41. Günther, A., Huth, V., Jurasinski, G. & Glatzel, S. The effect of biomass harvesting on greenhouse gas emissions from a rewetted temperate fen. *Glob. Change Biol. Bioenergy* **7**, 1092; 10.1111/gcbb.12214 (2015).

42. Clymo, R.S., Turunen, J. & Tolonen, K. Carbon accumulation in peatland. *Oikos* **81**, 368; 10.2307/3547057 (1998).

43. Young, D.M.*, et al.* Misinterpreting carbon accumulation rates in records from near-surface peat. *Sci. Rep.* **9**, 17939; 10.1038/s41598-019-53879-8 (2019).

44. Yu, Z., Loisel, J., Brosseau, D.P., Beilman, D.W. & Hunt, S.J. Global peatland dynamics since the last glacial maximum. *Geophys. Res. Lett.* **37**, n/a-n/a; 10.1029/2010GL043584 (2010).

45. Bockermann, C., Eickenscheidt, T. & Drösler, M. Greenhouse gas emissions and global warming potentials of five paludiculture plants in fen peatlands in southern Germany (2023).

46. Albert, M., Nagel, J., Schmidt, M., Nagel, R.-V. & Spellmann, H. Eine neue Generation von Ertragstafeln. *AFZ Der Wald*, **35-39**. https://www.nw-fva.de/fileadmin/nwfva/publikationen/pdf/albert_2022_eine_neue_generation_von_ertragstafeln.pdf (2022).

47. Thuille, A. & Schulze, E.-D. Carbon dynamics in successional and afforested spruce stands in Thuringia and the Alps. *Glob. Change Biol.* **12**, 325; 10.1111/j.1365-2486.2005.01078.x (2006).

48. Nagel, R., Meyer, P., Blaschke, M. & Feldmann, E. Strict forest protection: A meaningful contribution to Climate-Smart Forestry? An evaluation of temporal trends in the carbon balance of unmanaged forests in Germany. *Front. For. Glob. Change* **6;** 10.3389/ffgc.2023.1099558 (2023).

49. Krug, J.H.A. How can forest management increase biomass accumulation and CO2 sequestration? A case study on beech forests in Hesse, Germany. *Carbon Balance Manag.* **14**, 17; 10.1186/s13021-019-0132-x (2019).

50. Brandl, S., Paul, C., Knoke, T. & Falk, W. The influence of climate and management on survival probability for Germany’s most important tree species. *For. Ecol. Manag.* **458**, 117652; 10.1016/j.foreco.2019.117652 (2020).

51. Luderer, G.*, et al.* Ariadne-Report: Deutschland auf dem Weg zur Klimaneutralität 2045 – Szenarien und Pfade im Modellvergleich. https://ariadneprojekt.de/media/2022/02/Ariadne_Szenarienreport_Oktober2021_corr0222.pdf (2021).

52. CLC. CORINE Landcover database (2018).

53. Almena, A., Thornley, P., Chong, K. & Röder, M. Carbon dioxide removal potential from decentralised bioenergy with carbon capture and storage (BECCS) and the relevance of operational choices. *Biomass Bioenergy* **159**, 106406; 10.1016/j.biombioe.2022.106406 (2022).

54. Karlsson, S., Normann, F., Odenberger, M. & Johnsson, F. Modeling the development of a carbon capture and transportation infrastructure for Swedish industry. *Int. J. Greenhouse Gas Control* **124**, 103840; 10.1016/j.ijggc.2023.103840 (2023).

55. Lefvert, A. & Grönkvist, S. Smarter ways to capture carbon dioxide – exploring alternatives for small to medium-scale carbon capture in Kraft pulp mills. *Int. J. Greenhouse Gas Control* **127**, 103934; 10.1016/j.ijggc.2023.103934 (2023).

56. Cavallaro, N.*, et al.* Second state of the carbon cycle report (2018).

57. Global Carbon Project. Supplemental data of Global Carbon Project 2018 (2018).

58. Walker, W.S.*, et al.* The global potential for increased storage of carbon on land. *Proc. Natl. Acad. Sci. U. S. A.* **119**, e2111312119; 10.1073/pnas.2111312119 (2022).

59. NIR. Berichterstattung unter der Klimarahmenkonvention der Vereinten Nationen und dem Kyoto-Protokoll 2023: Nationaler Inventarbericht zum Deutschen Treibhausgasinventar 1990-2021. *Climate Change*, **28**. https://www.umweltbundesamt.de/sites/default/files/medien/11850/publikationen/28_2023_cc_berichterstattung_unter_der_klimarahmenkonvention.pdf (2023).

60. Böttcher, H.*, et al.* Options for strengthening natural carbon sinks and reducing land use emissions in the EU. https://www.oeko.de/fileadmin/oekodoc/Options_natural_sinks_EU.pdf (2021).

61. CAST. Climate change and greenhouse gas mitigation (Council for Agricultural Science and Technology, 2004).

62. Osterburg, B., Rüter, S., Freibauer, A. & et al. Handlungsoptionen für den Klimaschutz in der deutschen Agrar- und Forstwirtschaft. *Thünen Report*, **11**. http://nbn-resolving.de/urn:nbn:de:gbv:253-201312-dn052858-2 (2013).

63. Tanneberger, F.*, et al.* Saving soil carbon, greenhouse gas emissions, biodiversity and the economy: paludiculture as sustainable land use option in German fen peatlands. *Reg. Environ. Change* **22;** 10.1007/s10113-022-01900-8 (2022).

64. UBA. Submission under the United Nations Framework Convention on Climate Change and the Kyoto Protocol 2022 National Inventory Report for the German Greenhouse Gas Inventory 1990 – 2020. *Climate Change*, **25** (2022).

65. Wahren, A., Brust, K., Dittrich, I. & Edom, F. Regionalklima und Landschaftswasserhaushalt in *Paludikultur - Bewirtschaftung nasser Moore*, (ed. Wichtmann, W., Schröder, C. & Joosten, H.) 102 (Schweizerbart, 2016).

66. Joosten, H., Brust, K. & Couwenberg, J. MoorFutures® (Bundesamt für Naturschutz (BfN), 2015).

67. Närmann, F.*, et al.* Klimaschonende, biodiversitätsfördernde Bewirtschaftung von Niedermoorböden. https://www.bfn.de/sites/default/files/2021-11/Skript616.pdf (2021).

68. Nordt, A., Wichmann, S., Risse, J., Peters, J. & Schäfer, A. Potenziale und Hemmnisse für Paludikultur. https://www.dehst.de/SharedDocs/downloads/DE/projektmechanismen/Hintergrundpapier-hemmnisse-paludikultur.pdf?__blob=publicationFile&v=2 (2022).

69. Hanewinkel, M., Lessa Derci Augustynczik, A. & Yousefpour, R. Climate-smart forestry case study: Germany in *Forest Bioeconomy and Climate Change*, (ed. Hetemäki, L., Kangas, J. & Peltola, H.) 197 (Springer International Publishing, 2022).

70. BWaldG (1975).

71. Elsasser, P., Rock, J. & Rüter, S. Ein Vergleich unterschiedlicher Vorschläge zur Honorierung der Klimaschutzleistung der Wälder. *Thünen Working Paper*, **151**. https://literatur.thuenen.de/digbib_extern/dn062598.pdf (2020).

72. Merk, C., Andersen, G., Nordø, Å.D. & Helfrich, T. Carbon Capture and Storage - Publics in five countries around the North Sea prefer to do it on their own territory. *Kiel Working Paper*, **2252**. https://www.ifw-kiel.de/publications/carbon-capture-and-storage-publics-in-five-countries-around-the-north-sea-prefer-to-do-it-on-their-own-territory-31758/ (2023).

73. Sagebiel, J., Glenk, K. & Meyerhoff, J. Spatially explicit demand for afforestation. *For. Policy Econ.* **78**, 190; 10.1016/j.forpol.2017.01.021 (2017).

74. Hampicke, U., Küstner, A., Litterski, B. & Schäfer, A. Sukzessionswälder als Flächennutzungsalternative. https://www.dbu.de/OPAC/ab/DBU-Abschlussbericht-AZ-23880.pdf (2008).

75. Hupke, K.-D. Naturschutz (Springer Berlin Heidelberg, 2020).

76. Hampicke, U. Ökonomie und Naturschutz in *Handbuch Naturschutz und Landschaftspflege*, (ed. Hampicke, U., Böcker, R. & Konold, W.) 1 (Wiley, 2014).

77. UBA. Rahmendaten für den Projektionsbericht 2023. https://www.umweltbundesamt.de/sites/default/files/medien/479/publikationen/factsheet_rahmendaten_fuer_den_projektionsbericht_2023_fuer_deutschland.pdf (2022).

78. IEA. Bioenergy with Carbon Capture and Storage. https://www.iea.org/energy-system/carbon-capture-utilisation-and-storage/bioenergy-with-carbon-capture-and-storage (2023).

79. Schröder, J. & Naumann, K. Monitoring erneuerbarer Energien im Verkehr. (DBFZ, 2022).

80. EEG (2014/2023).

81. VCI. Die deutsche Chemie: Bereit für die Zukunft. https://www.vci.de/services/publikationen/vci-jahresbericht.jsp (2023).

82. Bethge, P. Kohlekraftwerke könnten künftig Holz verbrennen – gefördert mit Steuergeldern. *Spiegel Wissenschaft, (*2021).

83. Biofit. Bioenergy retrofits for Europe's industry. https://www.biofit-h2020.eu/publications-reports/BioFit_Final_Report.pdf (2022).

84. CarboFerro. CarboFerro - Entwicklung und Validierung eines innovativen Eisen-Kohlenstoff Präparates zur Gasreinigung und Effizienzsteigerung des Biogasprozesses. https://www.energetische-biomassenutzung.de/projekte-partner/details/project/show/Project/CarboFerro-727 (2023).

85. ReHydroPro. ReHydroPro - Regionale und energieautarke Produktion von grünem Wasserstoff durch direkte Kopplung eines Moduls zur Methanpyrolyse an eine Biogasanlage. https://www.energetische-biomassenutzung.de/projekte-partner/details/project/show/Project/ReHydroPro-724 (2023).

86. Thrän, D.*, et al.* The potential contribution of biogas to the security of gas supply in Germany. *Energ. Sustain. Soc. (Energy, Sustainability and Society)* **13;** 10.1186/s13705-023-00389-1 (2023).

87. Dotzauer, M.*, et al.* Kurzstudie zur Rolle von Biogas für ein klimaneutrales, 100 % erneuerbares Stromsystem 2035 (KS_BSKES). DBFZ and Wuppertal Institut. https://www.dbfz.de/fileadmin/user_upload/Referenzen/Studien/Kurzstudie_Biogas_2022.pdf (2022).

88. DBFZ. Umrüstung von Kohlekraftwerken auf Biomasse. https://www.dbfz.de/fileadmin/user_upload/Referenzen/Statements/2021_Position_Kohlekraftwerke.pdf (2021).

89. Bundesregierung. Evaluierungsbericht der Bundesregierung zum Kohlendioxid-Speicherungsgesetz (KSpG). https://www.bmwk.de/Redaktion/DE/Downloads/Energiedaten/evaluierungsbericht-bundesregierung-kspg.html (2022).

90. BÖR. Bioökonomie nachhaltig umsetzen! https://www.biooekonomierat.de/media/pdf/stellungnahmen/biooekonomierat-broschuere-nachhaltig-umsetzen-DE.pdf?m=1684941445& (2023).

91. Buss, W., Jansson, S., Wurzer, C. & Mašek, O. Synergies between BECCS and biochar—maximizing carbon sequestration potential by recycling wood ash. *Sustainable Chem. Eng.* **7**, 4204; 10.1021/acssuschemeng.8b05871 (2019).
